# Supplementary material for: Genomic landscape of adult testicular germ cell tumours in the 100,000 Genomes Project
Source: Nat Commun. 2024 Oct 26;15:9247. doi: 10.1038/s41467-024-53193-6 (PMC11513037; doi:10.1038/s41467-024-53193-6)
Supplement: Supplementary file 1 — Supplementary Information [file 41467_2024_53193_MOESM1_ESM.pdf]

Genomic landscape of adult testicular germ cell tumours in the 100,000 Genomes Project

Supplementary Methods

1. Clinical data 2
   1.1 Pathology reports 2
   1.2 Age distribution 2
   1.3 Pseudo ID system 3
2. DNA library preparation and sequencing 3
3. Driver identification 3
   3.1 Variant calling and filtering 3
   3.2 Mutation annotation 3
   3.3 Determining hypermutation status 4
   3.4 Protein-coding driver identification 4
      3.4.1 Pre-processing of input mutations 4
      3.4.2 Running driver identification methods 4
      3.4.3 Combination of driver identification methods 5
      3.4.4 Post-processing of candidate drivers 5
   3.5 Non-coding driver identification 6
      3.5.1 Defining sets of non-coding regions 7
      3.5.2 Detecting non-coding drivers 7
   3.6 OncoKB annotation 8
      3.6.1 Annotation of oncogenic mutations 8
      3.6.2 Lollipop plots of driver gene mutations 8
4. Analysis of copy number alterations (CNAs) 8
   4.1 Running CleanCNA 8
   4.2 Mutation clustering 10
      4.2.1 Running Varlap 10
   4.3 Identifying recurrent CNAs using GISTIC 10
      4.3.1 Preparing input copy number segmentation file 10
      4.3.2 Running GISTIC 10
      4.3.3 Prioritising likely gene targets of focal amplifications and deletions 11
   4.4 Extrachromosomal DNA detection 11
   4.5 Isochromosome classification 12
   4.6 Plackett-Luce-based ordering of CNAs 12
   4.7 Timing WGD 12
   4.8 Timing amplifications 13
5. Structural variants analysis 13
   5.1 Running SVclone 14
   5.2 Classifying structural variants 14
   5.3 SV annotation 15

5.4 Identifying SV hotspots 15
5.4.1 Relationship between genomic features and SV rates 15
5.4.2 Permuting SVs 16
5.4.3 Identifying SV hotspots 16
6. HLA analysis 17
6.1 HLA mutation calling with polysolver 17
6.2 HLA LOH calling with LOHHLA 18
6.3 HLA homozygosity 18
6.4 Neoantigen prediction 18

1. Clinical data

1.1 Pathology reports

All analyses were conducted within the Genomics England Research Environment (GERE) using Main Programme Data Release version 12. Primary genomic and clinical data from the 100,000 Genomes Project for each participant had been linked to available real-world clinical data collected by clinical teams at participant registration. In addition, secondary datasets collated by NHS Digital and Public Health England (PHE) were available, and include data on disease characteristics, treatment with systemic therapies and mortality.

All identifiable information had been removed from all datasets prior to release into the GERE, leaving anonymized records and 100,000 Genome Project participant identification numbers were used to facilitate linking of data.

In addition, anonymised histology reports were available from PHE for 53 participants and reports were reviewed by a Testicular Cancer GeCIP pathologist. A core dataset was obtained comprising clinical and pathological data including features such as baseline and follow up serum tumour markers (AFP, B-HCG, LDH), details of clinical stage at presentation, disease relapse and progression, chemotherapy received, tumour type, tumour size and the presence or absence of lymphovascular invasion in orchidectomy specimens. To define if serum tumour markers were raised or not, the presence of a normal AFP was a level of <10Ku/L (or equivalent in ng/ml) and a normal B-HCG was <2IU/L. Information relating to year of birth and NHS GMC for individual samples is not publicly available as this could compromise privacy and lead to identification of participants. Full tumour histologies are available in Supplementary Data 1. Separate written consent to publish additional clinical data was obtained from the family of participants with tumour ID GEL-TGCT-0056 following initial participant recruitment and consent.

1.2 Age distribution

We tested for bimodality using the `modetest` function obtained from the ‘multimode’ package[1]. The `locmodes` function was used to estimate the locations of modes and antimodes of distributions and their density values plotted in Supplementary Figure 1. Participants within the

seminoma subgroup were split into ‘young-onset’ and ‘old-onset’ using the mean age at diagnosis value (40 years of age).

1.3 Pseudo ID system

The Genomics England platekey identifiers assigned to sequenced samples and participant ID identifiers for individuals have exclusively remained within the secure GEL Research Environment, absent from both primary and supplementary materials. Instead, pseudo IDs (GEL-TGCT-****) have been consistently employed to reference individual-level data throughout the manuscript.

2. DNA library preparation and sequencing

Pre-extraction sample preparation and extraction of genomic DNA from germline blood and tumour fresh frozen tissue samples were carried out according to the specifications outlined in the Genomics England Sample Handling Guidance Documentation Version 4.0 (https://files.genomicsengland.co.uk/forms/Sample-Handling-Guidance-v4.0.pdf).

Sequencing and alignment was performed as described in the Genomics England Cancer Analysis Technical Information Document Version 1.11 (https://files.genomicsengland.co.uk/forms/Cancer-Analysis-Technical-Information-Document-v1-11-main.pdf). Samples were prepared using an Illumina TruSeq DNA Nano or TruSeq DNA PCR-Free preparation kit and then sequenced on a HiSeq X generating 150 bp paired-end reads. Sample cross-contamination checks were performed for germline and tumour samples as described in the Technical Information Document.

Reference bias in the Illumina Isaac (v03.16.02.19) aligner VAFs were corrected using FixVAF[2] (https://github.com/danchubb/FixVAF)

3. Driver identification

The approach used for cancer driver gene identification is described in detail below.

3.1 Variant calling and filtering

Small variant calling and filtering analyses were performed as described in the Methods section of the manuscript. Further detailed information about can be found in the Genomics England Cancer Analysis Technical Information Document Version 1.11 (https://files.genomicsengland.co.uk/forms/Cancer-Analysis-Technical-Information-Document-v1-11-main.pdf).

3.2 Mutation annotation

Somatic mutations were annotated to GRCh38 Ensembl v101 using the Ensembl Variant Effect Predictor (VEP)[3]. The following parameters were used: “vep -i <input_vcf> --assembly GRCh38 –no_stats –cache –offline –symbol –protein -o <output> --vcf –canonical –dir <ref_dir> --hgvs –hgvsg –fasta <GRCh38_fasta> --plugin CADD,<CADD_score_file> --plugin UTRannotator,<GRCh38_uORF_reference>”. The <CADD_score_file> was obtained using

CADD v1.6 (https://cadd.gs.washington.edu/)[4–6] with scores obtained for all SNV and indel mutations using the CAD software (https://github.com/kircherlab/CADD-scripts/), before being utilised by the VEP CADD plugin. The plugin "UTRannotator" (https://github.com/ImperialCardioGenetics/UTRannotator) was used to annotate the potential impact of five prime untranslated region (5' UTR) mutations[7].

3.3 Determining hypermutation status

Hypermutation status was assigned using the same criteria as in the Integrative OncoGenomics (IntOGen) pipeline (see section *Protein-coding driver identification*). Hypermutated samples were defined as those with more than 10000 mutations and with a mutation count exceeding Tukey's outlier condition, i.e., greater than 1.5 times the interquartile range (IQR) above the upper quartile (UQ) . Samples not generated via PCR-free library preparation were excluded.

3.4 Protein-coding driver identification

Protein-coding driver genes were identified using the IntOGen pipeline (https://bitbucket.org/intogen/intogen-plus/src/master/; downloaded February 2021; https://intogen.readthedocs.io/en/latest/)[8].

3.4.1 Pre-processing of input mutations

Somatic mutations passing the filtering criteria described previously across a tumour cohort were subject to initial sample and mutation pre-processing. In the case of multiple tumours from the same patient, the primary tumour was used (in the case of primary/recurrence pair), alternatively the tumour with the highest purity was used. Hypermutated tumours were flagged for exclusion from downstream driver gene identification if containing > 10,000 mutations and having an outlier mutation count (count > 1.5* interquartile range (IQR) + upper quartile (UQ)). Mutations found to be present in a Hartwig Panel of Normals were further excluded. Unless otherwise specified, mutations were mapped to canonical protein-coding transcripts from Ensembl v101.

3.4.2 Running driver identification methods

Seven driver gene identification methods were applied to both the GEL TGCT and The Cancer Genome Atlas (TCGA) TGCT cohorts as part of the IntOGen pipeline:

1. dNdSCV[9] (https://github.com/im3sanger/dndscv) is designed to detect genes under positive selection that show an excess of nonsynonymous (missense, nonsense, essential splice) mutations after correction for local trinucleotide context.
2. OncodriveFML[10] (https://bitbucket.org/bbglab/oncodrivefml/src/master/) aims to detect driver genes that show an enrichment of mutations with high functional impact. CADD[4–6] v1.6 scores were used as a measure of functional impact (https://cadd.gs.washington.edu/).
3. OncodriveCLUSTL[11] (https://bitbucket.org/bbglab/oncodriveclustl/src/master/) is a method designed to detect driver genes that are enriched for linear mutation clusters.

4. cBaSE[12] (http://genetics.bwh.harvard.edu/wiki/sunyaevlab/cbase) aims to detect driver genes under positive selection that exhibit a significant mutation count bias after correction by trinucleotide context.
5. MutPanning[13] (https://github.com/vanallenlab/MutPanningV2) is designed to detect driver genes that exhibit an enrichment of mutations with unusual nucleotide contexts compared to a background model.
6. HotMaps3D[14] (https://github.com/KarchinLab/HotMAPS) detects driver genes containing missense mutations that are spatially clustered together in the 3d structure of the protein. Protein structures obtained from the protein data bank (PDB) were downloaded in March 2020.
7. smRegions[15,16] (https://bitbucket.org/bbglab/smregions/src/master/) is designed to detect genes containing an enrichment of nonsynonymous mutations in regions of interest, such as protein domains, after correcting for trinucleotide context. This analysis utilised information from protein family (pfam) domains which were mapped to Ensembl v101 canonical transcripts.

3.4.3 Combination of driver identification methods

The driver combination procedure considered the top-40 ranked genes and their association *P* and *q*-values in each of the seven driver identification methods. Briefly, genes assigned as Tier 1 or Tier 2 somatically mutated genes in the COSMIC Cancer Gene Census[17] (https://cancer.sanger.ac.uk/census; v92 downloaded February 2021) were designated as "CGC" genes and represented a "truth" set of known drivers. Through comparison of the relative enrichment of known drivers in the top ranked gene lists a per-method weighting was obtained. Per-method ranked lists were combined using Schulze's voting method to generate a consensus ranking, with combined *P*-values estimated using a weighted Stouffer Z-score method.

Driver candidates were then classified into the following tiers:
Tier 1 – Candidates where the consensus ranking is higher than the ranking of the first gene with stouffer $q > 0.05$. These represent high confidence drivers.
Tier 2 – Candidates not meeting the criteria for Tier 1 but which are CGC genes, and show a combined stouffer $q_{CGC}<0.25$. Representing a "rescue" of known cancer drivers.
Tier 3 – Candidates not meeting the criteria for Tier 1 or Tier 2 but which have stouffer $q < 0.05$. These represent lower confidence drivers.
Tier 4 – Candidates not meeting criteria for Tier 1 or Tier 2 and stouffer $q > 0.05$. These represent candidates that are not likely to be drivers.

3.4.4 Post-processing of candidate drivers

Candidate driver genes were filtered on the basis of the following annotations:
1. "AUTOMATIC FAIL" – a candidate driver gene would be excluded from further consideration if annotated by at least one of the following:
    a. "TIER4" – categorised into Tier 4 by the combination procedure
    b. "1_METHOD" – only significant (Q<0.1) in 1/7 methods (non-CGC genes)

    c. “EXPRESSION” – gene has very low or absent expression in the relevant TCGA tumour type
    d. “OLFACTORY_RECEPTOR” – gene in list of olfactory receptor genes
    e. “KNOWN_ARTIFACT” – gene is in a known list of artifacts or long genes (e.g. TTN)
2. “MANUAL REVIEW” – if a gene is not excluded on the basis of any “AUTOMATIC FAIL” filters, it is retained as a candidate driver
    a. “GERMLINE” – non-Tier 1-CGC gene has 1+ mutations per sample and oe_syn/ms/lof > 1.5 based on GnomAD v2.1 constraint metric estimates (https://gnomad.broadinstitute.org/downloads#v2-constraint)
    b. “SAMPLE_3_MUTS” – non-CGC gene where there are 3+ mutations in 1+ tumour
    c. “LITERATURE” – non-CGC gene where there are no literature annotations according to CancerMine[18] (http://bionlp.bcgsc.ca/cancermine/; downloaded February 2021)
3. “AUTOMATIC PASS” – is not flagged by any “AUTOMATIC FAIL” or “MANUAL REVIEW” filters

Candidate driver roles were assigned on the basis of the dN/dS ratios for missense (w_{mis}) and nonsense (w_{non}) mutations for the given gene derived from dNdSCV (https://bitbucket.org/intogen/intogen-plus/src/master/core/intogen_core/postprocess/drivers/role.py):

- A “distance” metric was calculated by $distance = \frac{(wmis - wnon)}{\sqrt{2}}$
- Candidate drivers where $distance > 0.1$ represent those with an excess of missense to nonsense mutations and are assigned as “Oncogenes”
- Candidate drivers where $distance < 0.1$ represent those with an excess of nonsense to missense mutations and are assigned as “Tumour suppressor genes (TSGs)”
- Otherwise, the role of the candidate driver is unclear and is assigned as “Ambiguous”

In the case of multiple cohorts being run representing subsets of a given tumour type, a “consensus” role was designated comparing between each subtype role (“Oncogene” if 1+ cohort and no other cohorts assigned as “TSG”, “TSG” if in 1+ cohort and no other cohorts assigned as “Oncogene”, otherwise “ambiguous”).

Gene candidates were annotated by their overlap with any IntOGen cohorts from a previous 2020.02.01 pan-cancer analysis (https://www.intogen.org/download?file=IntOGen-Cohorts-20200201.zip) as well as from a pan-cancer TCGA analysis Bailey et al., 2018 (ref.[19]).

3.5 Non-coding driver identification

3.5.1 Defining sets of non-coding regions

Regions from candidate non-coding elements overlapping coding sequence (CDS) or exon regions from canonical protein-coding transcripts were removed using bedops[20] v2.4.39 (https://github.com/bedops/bedops).

The following sets of non-coding regions were defined:

1. Core promoters (n=19,283). Defined based on transcription start site (TSS) of canonical protein-coding transcripts: 200bp < TSS < 50bp. Coding sequence (CDS) regions were removed.
2. Distal promoters (n=19,296). Defined based on TSS of canonical protein-coding transcripts: 2kb < TSS. CDS regions were removed.
3. Five-prime untranslated regions (5'-UTRs; n=18,613). Defined based on canonical protein-coding transcripts. CDS regions were removed.
4. Three-prime untranslated regions (3'-UTRs; n=18,806). Defined based on canonical protein-coding transcripts. CDS regions were removed.
5. LincRNAs (n=16,510). Based on exon regions from transcripts annotated as lincRNAs in the ensembl v101 GTF. Exon regions from canonical protein-coding transcripts were removed.
6. miRNAs (n=1,793). Based on regions from transcripts annotated as miRNAs in the Ensembl v101 GTF. Exon regions from canonical protein-coding transcripts were removed.
7. Non-canonical splice regions (n=18,163). Defined from regions extending 30bp into the intron from essential splice donor or acceptor sites in canonical protein-coding transcripts. Exon regions from canonical protein-coding transcripts were removed.
8. Enhancers (n=130,996). Defined from Ensembl v101 regulatory elements annotated as "Enhancer". Exon regions from canonical protein-coding transcripts were removed.
9. Open chromatin regions (n=95,344). Defined from Ensembl v101 regulatory elements annotated as "Open chromatin". Exon regions from canonical protein-coding transcripts were removed.
10. CTCF sites (n=173,711). Defined from Ensembl v101 regulatory elements annotated as "CTCF sites". Exon regions from canonical protein-coding transcripts were removed.
11. TF binding sites (n=29,259). Defined from Ensembl v101 regulatory elements annotated as "TF binding sites". Exon regions from canonical protein-coding transcripts were removed.

3.5.2 Detecting non-coding drivers

OncodriveFML was run on sets of non-coding regions according to the following amended parameters from the protein-coding analysis: "indel-max" – indels are treated as a set of substitutions, with the functional impact of the indel mutation being the maximum of all the substitutions, and the background simulated as substitutions. A q<0.01 threshold was taken to indicate regions of significance.

3.6 OncoKB annotation
Nonsynonymous mutations in the 682 gene transcripts considered by OncoKB[21] v3.3 were annotated using the OncoKB API (https://www.oncokb.org/). In the first instance, the HGVSg identifier was used, however in rare instances where this failed a combination of gene symbol, consequence and HGVSp were used to map mutations to OncoKB annotations.

3.6.1 Annotation of oncogenic mutations

Nonsynonymous mutations in candidate driver genes were annotated as "Oncogenic" if either of the following criteria were met:

1. The mutation is annotated by OncoKB as "Oncogenic", "Likely Oncogenic" or "Predicted Oncogenic"
2. The driver role is "Oncogene", consequence is "missense" and mutation is recurrent (seen in 3+ tumours in cohort)
3. The driver role is "TSG" or "ambiguous" and either
   a. Consequence is protein-truncating ("splice acceptor", "splice donor", "frameshift", "stop lost", "stop gained", "start lost")
   b. Consequence is "missense" and mutation is recurrent (seen in 3+ tumours in cohort)

Nonsynonymous mutations not meeting these criteria were considered as a variant of uncertain significance (VUS).

3.6.2 Lollipop plots of driver gene mutations

Lollipop plots of driver gene mutations were generated using the R package trackViewer (https://github.com/jianhong/trackViewer)[22]. Pfam protein domains mapping to the ensembl v101 canonical transcripts were plotted. The protein position was taken from the first position in the HGVSp annotation, other than for splice donor and acceptor mutations where the codon nearest to the HGVSc transcript position was assigned as the protein position.

4. Analysis of copy number alterations (CNAs)

Copy number analyses were performed as described in the Methods section. Further details are provided below.

4.1 Running CleanCNA
Samples were classified as diploid or tetraploid according to its current CNA profile by first calculating ψ_t, a measure of average tumour ploidy, which is defined below.

$$\psi_t = \frac{\sum_{i=1}^{S} L_i (C_i^{Maj} + C_i^{Min})}{\sum_{i=1}^{S} L_i}$$

where, S is the number of copy number genome segments, and C_i^{Maj} and C_i^{Min} are the major and minor allele copy numbers for genome segment i, and L_i is the base pair length of genome segment i. Tumours were classified as having undergone whole genome duplication (WGD) if $2.9 - 2H < \psi_t$, where H is the fraction of the genome with a minor allele copy number of 0, and as non-WGD if this condition was not met.

B. Battenberg algorithm (version 2.2.7) requirements for a PASS:

(B1) All chromosomes have a copy number status.

(B2) Each chromosome length is at least 50% of the total chromosome length (using the appropriate genome build as reference).

(B3) No single homozygous deletion exceeds 10MB in length, else the cell is potentially unviable.

(B4) The fraction of a diploid genome with subclones at close to 50% (between 0.45-0.55) does not exceed 0.15 in combination with B3 (else the profile may be better called as tetraploid).

(B5) The fraction of a tetraploid genome in an odd copy number state [clonal 1:0,2:1,3:2] is not less than 0.15; else the profile may be better called as diploid.

C. CNAqc requirements for a PASS:

(C1) Re-estimation of sample purity using peaks of SNV distributions across the set of karyotypes that differs from the purity of the current copy number call by <5%.

(C2) Where a sample is called as tetraploid, that a peak of mutations exists within 5% VAF at copy number 2:2, and that this peak contains mutations at multiplicity 1 and 2.

D. DPClust (version 2.2.5) requirements for a PASS:

(D1) A peak of clonal (0.95-1.05 CCF) mutations exists.

(D2) No peak of superclonal mutations (>1.1 CCF) containing >5% total mutations for that sample exists.

(D3) No peak of mutations exists between 0.45-0.55 CCF that contains >5% total mutations in combination with A4.

We then summarised these requirements for a PASS across Battenberg, CNAqc, and DPClust into a consensus set of filters, namely (1) All chromosomes are included and have a copy number status, (2) Every chromosome has a copy number call across a sufficient proportion of its full length, (3) There is no excess of homozygous deletions present in the profile, (4) There is a peak of somatic SNVs at CCF 1, (5) There is no significant peak of somatic SNVs at CCF > 1, (6) The estimation of purity from CNAqc differs by no more than 5% from the purity estimate in the current profile.

4.2 Mutation clustering

SNVs and indels were grouped into clonal and subclonal clusters based on their CCF using the Bayesian Dirichlet process-based clustering algorithm DPClust[23] v2.2.5 (https://github.com/Wedge-Oxford/dpclust). Clusters (identified as local peaks in the posterior mutation density) were defined as clonal and subclonal according to their CCF peaks. Within individual samples, mutations were annotated as clonal if they were assigned to the cluster with CCF of approximately 1 and subclonal if assigned to a cluster with CCF<1. Clusters with less than 1% of mutations assigned were removed. Multidimensional Bayesian Dirichlet process-based clustering, implemented with DPClust[23] v2.2.5, was used to identify truncal, clonal and subclonal mutation clusters based on the CCF of the union list of somatic SNVs and indels across all 4 samples from the same primary tumour (GEL-TGCT-0058, regions A-D). SNVs were annotated as truncal when they were clonal across all samples from a participant.

4.2.1 Running Varlap

Varlap (https://github.com/bjpop/varlap) was used to carry out additional quality control of variants clustered with multidimensional DPClust. SNVs and indel quality was evaluated using the following metrics: tumour all/alt average mapping quality, tumour all/alt average number of mismatches in overlapping reads (NM), tumour all/alt average clipped bases, tumour all/alt average base quality, tumour all/alt align length.

4.3 Identifying recurrent CNAs using GISTIC

Below we describe steps for using GISTIC to identify recurrent CNAs in our study.

4.3.1 Preparing input copy number segmentation file

For every tumour passing copy number quality control measures, a copy number segmentation file as input for GISTIC2 (version 2.0.2.3) from Battenberg per-tumour segmentation output. From each copy number segment identified by Battenberg, the chromosomal coordinates, major ($nMaj$) and minor ($nMin$) copy number calls were obtained. In the case of subclonal copy number segments, $nMaj$ and $nMin$ were taken from the subclone with the largest tumour fraction.

Per-segment normalised copy number was calculated by:

$$SegCN = log_2(nMaj + nMin) - log_2(average\_CN)$$

Where $average\_CN$ was taken as the average copy number across the tumour rounded to the nearest whole integer.

Copy number at chrX was normalised by:

$$SegCN = log_2(nMaj + nMin) - log_2\left(\frac{averageCN}{2}\right)$$

4.3.2 Running GISTIC

GISTIC2[24] v2.0.2.3 was run to identify recurrent arm-level copy number events, as well as focal amplifications and deletions (https://github.com/broadinstitute/gistic2). The following parameters were used: "-conf 0.99 -broad 1 -qvt 0.25 -genegistic 1 -gcm extreme -brlen 0.5 -rx 0 -twoside 1 -scent median -armpeel 1 -arb 1 -refgene hg38.UCSC.add_miR.160920.refgene.mat".

4.3.3 Prioritising likely gene targets of focal amplifications and deletions

Candidate target genes at focal amplifications and deletions were annotated according to the following criteria:

1. Overlap with genes at focal amplifications and deletions previously reported in a pan-cancer TCGA study as found in Supplementary Table 3 of Zack et al. (ref.[25]). Comparisons were made both with the overall pan-cancer GISTIC2 analysis, as well as GISTIC2 analysis restricted to the given tumour type. Special consideration was given to genes specifically highlighted in the Zack et al. (ref.[25]) analysis as being prioritised candidates
2. Overlap with Cosmic Cancer Gene Census genes and whether their annotated role (Oncogene (OG)/tumour suppressor gene (TSG)/ambiguous) is consistent with the copy number change (OG with amplifications, TSG with deletions)
3. Overlap with driver genes identified as being significantly mutated in this study, and whether the driver's likely role (Oncogene (OG)/tumour suppressor gene (TSG)/ambiguous) is consistent with the copy number change (OG with amplifications, TSG with deletions)

On the basis of the above criteria, "consensus" driver genes were manually assigned to peaks. To maximise detection of overlapping genes, comparisons were made with all potential gene synonyms as available from the HUGO gene nomenclature name committee (https://www.genenames.org/).

4.4 Extrachromosomal DNA detection

Potential extrachromosomal DNA (ecDNA) molecules were detected from tumour bam files using AmpliconArchitect[26] v1.2 (https://github.com/virajbdeshpande/AmpliconArchitect). Briefly, per-tumour "seed" regions were prepared from Battenberg copy-number segmentation output if a segment was >100kb and the total copy number was > 5. AmpliconArchitect was then run using these "seed" regions to extract overlapping sequence reads from the tumour bamfile and construct candidate amplicons.

Candidate amplicons were classified using AmpliconClassifier v0.4.6 (https://github.com/jluebeck/AmpliconClassifier) into the following categories:

1) Cyclic (truly circularised ecDNA)
2) Complex non-cyclic
3) Linear amplification
4) No amp/invalid. Amplicons were highlighted if containing a known highly amplified oncogene (*MDM2*, *MYC*, *EGFR*, *CDK4, ERBB2, SOX2, TERT, CCND1, E2F3, CCNE1, CDK6,*

MDM4, NEDD9, MCL1, AKT3, BCL2L1, ZNF217, KRAS, PDGFRA, AKT1, MYCL, NKX2-1, IGF1R, PAX8; as per ref.[27]).

4.5 Isochromosome classification
Canonical isochromosomes were identified using Battenberg output on the basis that at least two excess copies of 12p relative to 12q were gained through the centromere. Where p and q arms were segmented, then the requirement became that there were at least two excess copies of the largest 12p segment relative to the largest 12q segment.

4.6 Plackett-Luce-based ordering of CNAs
Mutation-drivers and enriched CNAs were incorporated into a timing model based on their clonality. Three types of CNA were considered: gain, loss of heterozygosity (LOH), and homozygous deletion (HD). To include only recurrent or enriched regions, CNA events of each type were piled up across all samples to get the frequency landscape of each CNA type based on all observed breakpoints. Next, a permutation test (n = 1,000) followed by false discovery rate (FDR)-based multiple-testing correction was undertaken to identify regions that were significantly enriched above the random background CNA rate. The same approach was used to identify regions significantly suppressed or depleted below the random background CNA rate. Enriched regions encompassing the HLA region (6p21), specific to telomeric ends, present as singletons or occurring on the short-arm of acrocentric chromosomes were excluded.

Battenberg copy number calls were used to assign the clonality of CNAs. SNVs and indels were classified as clonal (CCF = 0.95) and subclonal (CCF < 1) using DPClust. For each mutational driver (with ≥5% recurrence), the CCF of each variant was estimated by adjusting the variant allele frequency according to the CNA status of the locus and purity of the tumour sample, as described previously[23]. All events were combined per sample and ordered based on the CCF. Where more than one tree could be inferred based on subclonal events, all possible trees were generated and randomly chosen in each iteration of the ordering events. To time the events based on the entire dataset, events were ordered based on clonality (randomized clonal events followed by a sampled tree of subclonal events) in each sample.

The estimated number of chromosomes bearing the mutation and major/minor copy number status were used to classify mutations and CNAs occurring pre-WGD and post-WGD. The Plackett Luce model[28,29] for ordering partial rankings was implemented (https://github.com/hturner/PlackettLuce) to infer the order of events based on the ordering matrix of the entire dataset while allowing for unobserved events. This analysis was undertaken for 1,000 iterations to obtain the 95% CI of the timing estimate of each event. We repeated this analysis across histological subtypes.

4.7 Timing WGD
To ensure each copy of a chromosome within a sample had sufficient coverage to accurately time clonal copy number changes along it, we limited the timing analysis to samples with an average number of reads per chromosome copy (nrpcc) ≥ 14 (**Supplementary Fig. 20**). The average nrpcc in a given sample was calculated using the following formula[30]:

$$Average\ reads\ per\ chromosome\ copy\ = \frac{purity}{(purity*ploidy) + ((1 - purity)*2) * tumour\ coverage}$$

The R package mutationtimeR[30] was used to identify clonal substitutions and estimate the probability of these occurring prior before a copy number gain.

To estimate the substitution burden prior to WGD, all 2+0, 2+1 and 2+2 copy number segments predicted to be involved in WGD were identified. Low confidence segments, defined as those with greater 0.5 width confidence intervals for the mutation time estimate of their gain, were excluded as per Oliver et al.[31]. Substitutions across these segments predicted to occur prior to duplication were summed and adjusted for the pre-duplication substitutions on the minor allele of 2+0 or 2+1 segments that cannot be identified[31]. The adjusted value was extrapolated to a genome-wide estimate, according to the number of bases covered by the copy number segments included. A previously published mutation rate range of 0.5-0.7 per haploid genome per cell division within primordial germ cells (PGCs) was used to convert substitution burden to a number of cell divisions[32]. An important assumption underlying this approach was that most detectable mutations emerged post-PGC specification and that the earliest embryonic mutations are not detectable. Late WGD cases (GEL-TGCT-0016, GEL-TGCT-0043, GEL-TGCT-0012) were identified based on the number of substitutions estimated to have occurred prior to WGD.

4.8 Timing amplifications

AmplificationTimeR version 0.0.1.0 (https://github.com/Wedge-lab/AmplificationTimeR), An R package for timing sequential amplification events, was used to infer the event order of whole genome duplication and enriched high level gains observed on chromosome 12 (chr12:55549-34716255). The relative ordering of events was inferred based on the highest copy number and multiplicity of mutations (see section *Running CleanCNA*). The model uses all mutations found in a segment and assumes that the mutation rate is constant. Further, as part of AmplificationTimeR, we calculated timings using only C>T mutations at CpG dinucleotides as a proxy for clock-like mutations (see section *Mutational Signatures Analysis* in Methods) as the model assumes constant mutation rate.

5. Structural variants analysis

Structural variant analyses were performed as described in the Methods section. Further details are provided below.

Somatically acquired long interspersed nuclear element (LINE-1) retrotransposition events were identified using xTea[33](https://github.com/parklab/xTea). Other retrotransposition categories, including Alu elements, SINE-VNTR-Alu elements and processed pseudogene, collectively comprise ≤3% of retrotransposition events across human cancers[34] and were therefore not considered. Retrotransposition events are mechanistically distinct from other SV-generating events[34] and retrotransposition events were therefore excluded from our SV analyses, as per Li et al. (ref.[35]). SVs identified using the graph-based consensus approach were classified as part of a retrotransposition event and excluded if: (1) xTea identified a transduced region within 10kb

of either SV break point in the same sample, or (2) xTea identified a transduced region within 10kb of either SV break point in at least one other testicular samples. A 10kb threshold was chosen as the majority of somatically acquired transductions span regions <10kb from a canonical LINE-1 element[36].

5.1 Running SVclone

SVclone (https://github.com/mcmero/SVclone) was applied to GEL samples using an approach similar to that described in Dentro et al. (ref.[37]). A detailed explanation of the SVclone algorithm and pipeline can be found in Cmero et al. (ref.[38]).

Using the final SV vcf, SNV vcf, Battenberg copy number profile and purity estimate, the following preprocessing steps were carried out using SVclone:

- *annotate* - each of the SV pairs was annotated as coming from a deletion, inversion, translocation, or duplication
- *count* - normal and variant read counts for each SV were extracted from the BAM file and adjusted based on SV event type
- *filter* - the following filters were applied:
    - any SV or SNV that was not matched to a valid copy number segment was removed
    - any SV that lacked split or spanning supporting reads was removed
    - any SV less than 1000bp was removed

BAM file read length, insert length and standard deviation of insert length were inferred dynamically using SVclone. Mean BAM coverage was 95 and maximum considered copy-number was 10. The CCF of SNVs and SVs was simultaneously clustered and determined using the co-clustering mode of SVclone. MCMC was carried out for 25,000 iterations to approximate the posterior distributions over model parameters. SVclone's default approach was used to determine cluster membership for the variants used during clustering, and cluster mean CCFs. Variants not used in clustering were retroactively assigned to the most likely cluster using SVclone's post_assign procedure. Low-count clusters were filtered out (< 10 variants or < 1% of variants) and variants from these clusters were re-assigned to the second most likely cluster.

5.2 Classifying structural variants

Rearrangements identified by the graph-based consensus approach were grouped into footprints and clusters based on their proximity within the genome, the overall number of events in the genome, and the size of these events, using ClusterSV[35]. Rearrangement footprints represent sets of rearrangement breakpoints that are positionally associated, whilst rearrangement clusters represent sets of rearrangements that are mechanistically associated. Rearrangement footprints were described using the string approach proposed by Li et al[35]. Rearrangement clusters were classified as being a simple event (deletion, tandem duplication, balanced inversion, balanced translocation, unbalanced translocation, or simple unclassified) or a complex event (chromoplexy, chromothripsis, or complex unclassified). Simple and complex events were defined as clusters comprising ≤2 or ≥3 individual rearrangements respectively.

Chromothripsis events were inferred using established criteria[39,40]. A rearrangement cluster was defined as chromothripsis if it met all the following criteria:
• A contiguous series of four genome segments oscillating between two copy number states, or five genome segments oscillating between three copy number states.
• At least six interleaved intra-chromosomal rearrangements, as per Cortes-Ciriano et al.[40].
• No evidence (false discovery rate > 0.2) that the distribution of intra-chromosomal fragment join orientations diverge from a multinomial distribution with equal probabilities for each of the four orientation categories (duplication-like, deletion-like, head-to-head inversion, and tail-to-tail inversion).

A rearrangement cluster was defined as chromoplexy if it met all the following criteria:
• Contains a chain of rearrangements spanning at least three chromosomes[35]. SV chains were identified using a graph-based approach, in which nodes represent breakpoints, and are connected by an edge if they are not involved in the same rearrangement and fall within 1Mb of each other. Graph-based approach implemented using the igraph R-package (v1.2.4.2)[41].
• At least 50% of rearrangement footprints in the cluster represent balanced translocations, either with no observed copy number change, or a deletion bridge between the break ends.
• Consists of between 3 and 30 rearrangements.

5.3 SV annotation
The final set of high-quality structural variant (SV) calls were annotated by SNPeff[42] v5.1 (http://pcingola.github.io/SnpEff/) according to GRCh38 Ensembl v101 reference to identify their likely effect on gene transcript and/or protein function. Annotations were restricted to canonical protein-coding transcripts in Ensembl v101. These annotated SV calls were further prioritized using “simple sv annotation” (https://github.com/AstraZeneca-NGS/simple_sv_annotation) to highlight exon-level deletion events in candidate driver genes.

5.4 Identifying SV hotspots
Rates of somatic structural variation differ throughout the genome and are influenced by local genomic features[35]. Genome regions enriched for simple SVs were identified using a permutation-based approach that considered genomic features associated with structural variation occurrence, as per Glodzik et al. (ref.[43]) and as described below. Deletions, tandem duplications, balanced inversions, balanced inter-chromosomal translocations, and unclassified simple SVs were considered separately. Individual rearrangements that form parts of complex SVs were excluded from this analysis. Seminomas and non-seminomas were also analysed separately.

The steps described below were performed as part of the pipeline used to identify SV hotspots.

5.4.1 Relationship between genomic features and SV rates

Negative binomial regression was used to test associations between genomic features and numbers of SVs of each simple class[43]. The following features were included in the models:

average total copy number across the bin in the TGCT sample set, GC content, the presence of genes highly or lowly expressed in TGCT, ALU repeats, other genomic repeats, segmental duplications, fragile sites, replication timing, and DNase, H3K36me3 and H3K9me3 peaks[43]. Highly and lowly expressed genes were defined as those with mean RSEM value in the top 25% and bottom 75% of protein-coding genes in TCGA samples with RNA-Seq (TCGA, 2012). ALU and other genomic repeats were obtained from the UCSC Genome Browser[44]. Segmental duplications were obtained for GRCh38 from the Segmental Duplication Database (https://humanparalogy.gs.washington.edu)[45]. Fragile sites were obtained from Bignell et al. (ref. [46]). Replication timing data from a human embryonic stem cell line (BG01) were obtained from ReplicationDomain[47]. DNAse-seq data (ENCFF589CRI) and ChIP-seq data for histones H3K36me3 (ENCFF437RZA) and H3K9me3 (ENCFF010BPD) were obtained for adult human testis tissue from ENCODE (The ENCODE Project Consortium, et al., 2020).

5.4.2 Permuting SVs

SVs were simulated to test whether the number of SVs observed in a region was greater than expected by chance given the local genomic features[43]. SVs were simulated for each simple SV class, preserving the number and length (distance between intra-chromosomal SV break ends) of SVs observed in the TGCT sample sets. To simulate SVs, the genome was divided into non-overlapping 1Mb bins and the genomic features (listed above) of each bin summarized. All genomic features were normalized to a mean of 0 and standard deviation of 1 to aid comparisons. The number of break ends expected in each bin was then estimated using the effect estimates from the previously generated negative binomial regression model. For each observed SV, an SV was simulated by sampling a bin under probabilities proportional to the expected numbers of break ends in each bin. For intra-chromosomal SVs, a partner break end was then simulated by selecting the position either upstream or downstream (with equal probability) equal in distance to the distance between the two break ends in the observed SV. For inter-chromosomal SVs, a partner break end was simulated by sampling a bin under probabilities proportional to the expected numbers of break ends in each bin, excluding bins on the same chromosome. SVs were re-simulated if either break end fell within an uncallable region (a telomere or centromere). SVs were simulated 1,000 times to generate a null distribution of expected SV numbers for the 1Mb bins.

5.4.3 Identifying SV hotspots

Piece-wise constant fitting (PCF) was used to identify regions of the genome containing greater numbers of SV break ends than expected[43]. SV break ends were first sorted by position and the distance between successive break ends calculated. PCF was then applied to the $\log_{10}$ of these inter-mutational distances (IMD). SV hotspots were identified by first computing the observed (d_i^{obs}) and expected (d_i^{exp}) number of breakends per base pair for each PCF segment (*i*):

$$d_i^{obs} = \frac{a_i}{s_i}$$

$$d_i^{exp} = \frac{\sum_{j=1}^{n} b_j}{ns^{bin}}$$

Where a_i is the number of break ends in the segment, s_i is the length of the segment in base pairs, n is the number of bins overlapping the segment, b_j is the expected number of SVs in bin j, and s^{bin} is the bin size (1Mb). A simple SV enrichment factor (β_i^{simple}) is then computed for each PCF segment as:

$$\beta_i^{simple} = \frac{d_i^{obs}}{d_i^{exp}}$$

The PCF algorithm requires parameters γ (that controls the smoothness of the segmentation) and k_{min} (the minimum number of mutations in a segment). False discovery rates (FDRs) at each β^{simple} value were estimated by applying PCF to both the observed and simulated SV sets and dividing the mean number of segments with a β^{simple} value at least as great in the simulated SV sets by the number of segments with a β^{simple} value at least as great in the observed SV set. A maximum FDR of one was set and FDR values equal to zero were changed to the lowest non-zero FDR value observed. Optimal γ and k_{min} values were chosen by repeating this process for values of γ between 1 and 20, and values of k_{min} between 2 and 20, and selecting values that maximized the number of hotspots identified, whilst minimizing the FDR. In the final analysis γ=10 was used throughout, whilst k_{min}= 4 was used for all structural variant classes. SV hotspots where no SVs were supported by CNAs were considered potential artifacts and removed.

6. HLA analysis

6.1 HLA mutation calling with polysolver

Somatic mutations in the HLA locus were predicted using polysolver[48]. First, alleles were converted into a polysolver-compatible format (lower case, digits separated by underscore) and outputted into a patient-specific winners.hla.txt file, following the standard output of polysolver. The same winners.hla.txt files were used as input for LOHHLA, with polysolver's comprehensive deduplicated fasta of HLA haplotype sequences as reference. Then, the mutation detection script of polysolver (shell_call_hla_mutations_from_type) was run on matched tumour-normal pairs to call tumour-specific single nucleotide alterations in HLA-aligned sequencing reads using MuTect (v1.16). In addition, Strelka2 (v2.9.9)[49] was run to detect short insertions and deletions in HLA-aligned reads as this version offers increased sensitivity over polysolver's default caller. Finally, both single nucleotide mutations and indels passing quality control were annotated by polysolver's built-in annotation script, shell_annotate_hla_mutations. Samples with at least one protein-changing mutation in an HLA gene were labelled as having "HLA mutation". 4 samples had a haplotype not contained in the sequence database of Polysolver. For these samples, HLA mutation and LOH calling (see section *HLA LOH calling with LOHHLA*) was restricted to the remaining five compatible haplotypes. Lastly, we used variant calling output (see section *Variant calling and filtering* in Methods) to identify nonsynonymous/frameshift mutations in a set of

antigen presenting genes (*PSME3*, *PSME1*, *PSME4*, *PSME2*, *ERAP2*, *TAP2*, *ERAP1*, *HSPBP1, PDIA3, CALR, B2M, PSMA7, IRF1, CANX, TAP1, CIITA).*

6.2 HLA LOH calling with LOHHLA

HLA LOH calling with LOHHLA[50] was performed as described in the Methods section. LOH could not be evaluated for two samples that had highly similar haplotypes in all HLA A/B/C genes; in GEL-TGCT-0057, all HLA haplotypes are highly similar, with not enough distinguishing positions; in GEL-TGCT-0047, HLA-A/B are highly similar and HLA-C had a haplotype incompatible with polysolver.

6.3 HLA homozygosity

HLA homozygosity was ascertained using raw HLA predictions, whereby homozygosity supertypes were assigned to TGCT samples. These supertypes are only defined for HLA-A and HLA-B.

6.4 Neoantigen prediction

We predicted neoantigens using NeoPredPipe, a python-based pipeline combining Annovar and netMHCpan 4.0[51]. Briefly, all quality-controlled somatic mutations were annotated, and for all non-synonymous exonic mutations the mutated peptide sequence was predicted. We took any 8-11-mer spanning the mutated amino acid(s), resulting in either (i) a 21-aa window for SNVs or (ii) a peptide until the next predicted stop codon for FSs. These peptides were evaluated according to their novelty and predicted binding strength to the patient's six-allele HLA set. Peptides that appear novel when compared to the healthy human proteome with binding rank 2 and below (amongst the best 2% of binders compared to a large set of random peptides) were reported as neoantigens. All patient-specific HLA alleles were used for neoantigen predictions, regardless of mutation or LOH status of the HLA locus. A mutation was considered neoantigenic if at least one of its downstream mutated peptides were a neoantigen with respect to any of the patient's six HLA alleles. Only mutations that passed all variant filters (Filter=PASS in the vcf) were considered. Neoantigen burden was defined as the total number of neoantigenic mutations in a sample.

Two samples that were not PCR-free were excluded from analysis, as these had an inflated mutation and consequently neoantigen count. The four multi-region biopsies showed a high concordance of neoantigens, and therefore we only included the highest purity sample in downstream analysis.

References

1. Ameijeiras-Alonso, J., Crujeiras, R. M. & Rodríguez-Casal, A. Mode testing, critical bandwidth and excess mass. *Test* **28**, 900–919 (2019).
2. Cornish, A. J. *et al.* Reference bias in the Illumina Isaac aligner. *Bioinformatics* vol. 36 4671–4672 (2020).

3. McLaren, W. *et al.* The Ensembl Variant Effect Predictor. *Genome Biol.* **17**, 122 (2016).

4. Kircher, M. *et al.* A general framework for estimating the relative pathogenicity of human genetic variants. *Nat. Genet.* **46**, 310–315 (2014).

5. Rentzsch, P., Witten, D., Cooper, G. M., Shendure, J. & Kircher, M. CADD: predicting the deleteriousness of variants throughout the human genome. *Nucleic Acids Res.* **47**, D886–D894 (2019).

6. Rentzsch, P., Schubach, M., Shendure, J. & Kircher, M. CADD-Splice-improving genome-wide variant effect prediction using deep learning-derived splice scores. *Genome Med.* **13**, 31 (2021).

7. Zhang, X., Wakeling, M., Ware, J. & Whiffin, N. Annotating high-impact 5'untranslated region variants with the UTRannotator. *Bioinformatics* **37**, 1171–1173 (2021).

8. Martínez-Jiménez, F. *et al.* A compendium of mutational cancer driver genes. *Nat. Rev. Cancer* **20**, 555–572 (2020).

9. Martincorena, I. *et al.* Universal patterns of selection in cancer and somatic tissues. *Cell* **171**, 1029–1041.e21 (2017).

10. Mularoni, L., Sabarinathan, R., Deu-Pons, J., Gonzalez-Perez, A. & López-Bigas, N. OncodriveFML: a general framework to identify coding and non-coding regions with cancer driver mutations. *Genome Biol.* **17**, 128 (2016).

11. Arnedo-Pac, C., Mularoni, L., Muiños, F., Gonzalez-Perez, A. & Lopez-Bigas, N. OncodriveCLUSTL: a sequence-based clustering method to identify cancer drivers. *Bioinformatics* **35**, 4788–4790 (2019).

12. Weghorn, D. & Sunyaev, S. Bayesian inference of negative and positive selection in human cancers. *Nat. Genet.* **49**, 1785–1788 (2017).

13. Dietlein, F. *et al.* Identification of cancer driver genes based on nucleotide context. *Nat. Genet.* **52**, 208–218 (2020).

14. Tokheim, C. *et al.* Exome-Scale Discovery of Hotspot Mutation Regions in Human Cancer

Using 3D Protein Structure. *Cancer Res.* **76**, 3719–3731 (2016).

15. Porta-Pardo, E. & Godzik, A. e-Driver: a novel method to identify protein regions driving cancer. *Bioinformatics* **30**, 3109–3114 (2014).
16. Martínez-Jiménez, F., Muiños, F., Lopez-Arribillaga, E., Lopez-Bigas, N. & Gonzalez-Perez, A. Disruption of ubiquitin mediated proteolysis is a widespread mechanism of tumorigenesis. *bioRxiv* 507764 (2018) doi:10.1101/507764.
17. Sondka, Z. *et al.* The COSMIC Cancer Gene Census: describing genetic dysfunction across all human cancers. *Nat. Rev. Cancer* **18**, 696–705 (2018).
18. Lever, J., Zhao, E. Y., Grewal, J., Jones, M. R. & Jones, S. J. M. CancerMine: a literature-mined resource for drivers, oncogenes and tumor suppressors in cancer. *Nat. Methods* **16**, 505–507 (2019).
19. Bailey, M. H. *et al.* Comprehensive Characterization of Cancer Driver Genes and Mutations. *Cell* **174**, 1034–1035 (2018).
20. Neph, S. *et al.* BEDOPS: high-performance genomic feature operations. *Bioinformatics* **28**, 1919–1920 (2012).
21. Chakravarty, D. *et al.* OncoKB: A Precision Oncology Knowledge Base. *JCO Precis Oncol* **2017**, (2017).
22. Ou, J. & Zhu, L. J. trackViewer: a Bioconductor package for interactive and integrative visualization of multi-omics data. *Nat. Methods* **16**, 453–454 (2019).
23. Bolli, N. *et al.* Heterogeneity of genomic evolution and mutational profiles in multiple myeloma. *Nat. Commun.* **5**, 2997 (2014).
24. Mermel, C. H. *et al.* GISTIC2.0 facilitates sensitive and confident localization of the targets of focal somatic copy-number alteration in human cancers. *Genome Biol.* **12**, R41 (2011).
25. Zack, T. I. *et al.* Pan-cancer patterns of somatic copy number alteration. *Nat. Genet.* **45**, 1134–1140 (2013).
26. Deshpande, V. *et al.* Exploring the landscape of focal amplifications in cancer using

AmpliconArchitect. *Nat. Commun.* **10**, 392 (2019).

27. Kim, H. *et al.* Extrachromosomal DNA is associated with oncogene amplification and poor outcome across multiple cancers. *Nat. Genet.* **52**, 891–897 (2020).
28. Plackett, R. L. The analysis of permutations. *J. R. Stat. Soc. Ser. C Appl. Stat.* **24**, 193 (1975).
29. Duncan Luce, R. *Individual Choice Behavior: A Theoretical Analysis*. (Courier Corporation, 2012).
30. Gerstung, M. *et al.* The evolutionary history of 2,658 cancers. *Nature* **578**, 122–128 (2020).
31. Oliver, T. R. W. *et al.* Clonal diversification and histogenesis of malignant germ cell tumours. *Nat. Commun.* **13**, 4272 (2022).
32. Rahbari, R. *et al.* Timing, rates and spectra of human germline mutation. *Nat. Genet.* **48**, 126–133 (2016).
33. Chu, C. *et al.* Comprehensive identification of transposable element insertions using multiple sequencing technologies. *Nat. Commun.* **12**, 1–12 (2021).
34. Rodriguez-Martin, B. *et al.* Pan-cancer analysis of whole genomes reveals driver rearrangements promoted by LINE-1 retrotransposition in human tumours. *bioRxiv* 179705 (2017) doi:10.1101/179705.
35. Li, Y. *et al.* Patterns of somatic structural variation in human cancer genomes. *Nature* **578**, 112–121 (2020).
36. Tubio, J. M. C. *et al.* Mobile DNA in cancer. Extensive transduction of nonrepetitive DNA mediated by L1 retrotransposition in cancer genomes. *Science* **345**, 1251343 (2014).
37. Dentro, S. C. *et al.* Characterizing genetic intra-tumor heterogeneity across 2,658 human cancer genomes. *Cell* **184**, 2239–2254.e39 (2021).
38. Cmero, M. *et al.* Inferring structural variant cancer cell fraction. *Nat. Commun.* **11**, 730 (2020).
39. Korbel, J. O. & Campbell, P. J. Criteria for inference of chromothripsis in cancer genomes.

Cell **152**, 1226–1236 (2013).

40. Cortés-Ciriano, I. *et al.* Comprehensive analysis of chromothripsis in 2,658 human cancers using whole-genome sequencing. *Nat. Genet.* **52**, 331–341 (2020).
41. Csardi, G., Nepusz, T. & Others. The igraph software package for complex network research. *InterJournal, complex systems* **1695**, 1–9 (2006).
42. Cingolani, P. *et al.* A program for annotating and predicting the effects of single nucleotide polymorphisms, SnpEff: SNPs in the genome of Drosophila melanogaster strain w1118; iso-2; iso-3. *Fly* **6**, 80–92 (2012).
43. Glodzik, D. *et al.* A somatic-mutational process recurrently duplicates germline susceptibility loci and tissue-specific super-enhancers in breast cancers. *Nat. Genet.* **49**, 341–348 (2017).
44. Haeussler, M. *et al.* The UCSC Genome Browser database: 2019 update. *Nucleic Acids Res.* **47**, D853–D858 (2019).
45. She, X. *et al.* Shotgun sequence assembly and recent segmental duplications within the human genome. *Nature* **431**, 927–930 (2004).
46. Bignell, G. R. *et al.* Signatures of mutation and selection in the cancer genome. *Nature* **463**, 893–898 (2010).
47. Weddington, N. *et al.* ReplicationDomain: a visualization tool and comparative database for genome-wide replication timing data. *BMC Bioinformatics* **9**, 530 (2008).
48. Shukla, S. A. *et al.* Comprehensive analysis of cancer-associated somatic mutations in class I HLA genes. *Nat. Biotechnol.* **33**, 1152–1158 (2015).
49. Kim, S. *et al.* Strelka2: fast and accurate calling of germline and somatic variants. *Nat. Methods* **15**, 591–594 (2018).
50. McGranahan, N. *et al.* Allele-Specific HLA Loss and Immune Escape in Lung Cancer Evolution. *Cell* **171**, 1259–1271.e11 (2017).
51. Schenck, R. O., Lakatos, E., Gatenbee, C., Graham, T. A. & Anderson, A. R. A.

NeoPredPipe: high-throughput neoantigen prediction and recognition potential pipeline. *BMC Bioinformatics* **20**, 264 (2019).

Genomic landscape of adult testicular germ cell tumours in the 100,000 Genomes Project

Supplementary Figures

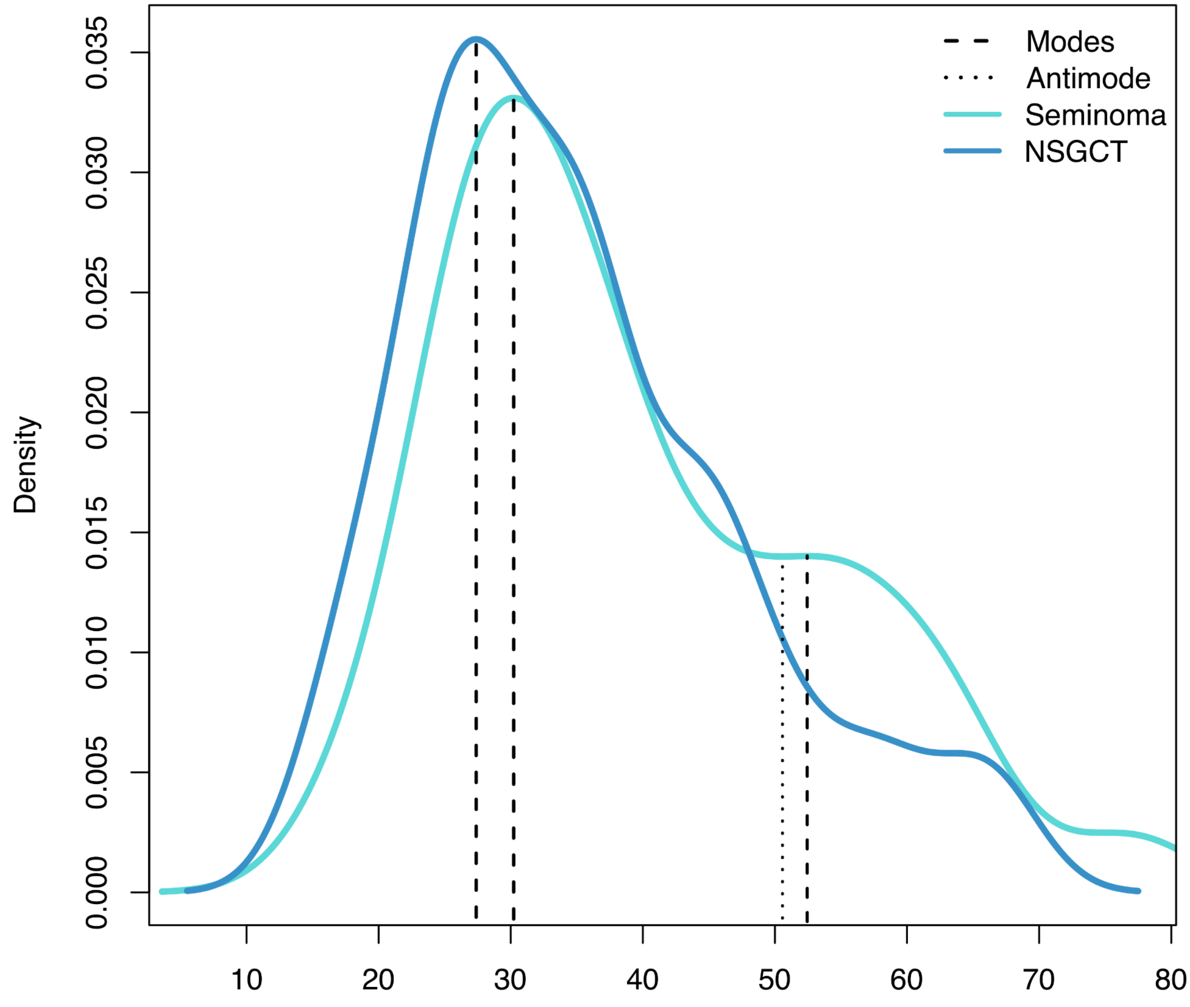

Supplementary Figure 1. Modeling age distribution in GEL TGCT cohort using the R package multimode. Separate fits are shown for seminoma and NSGCT subtypes.

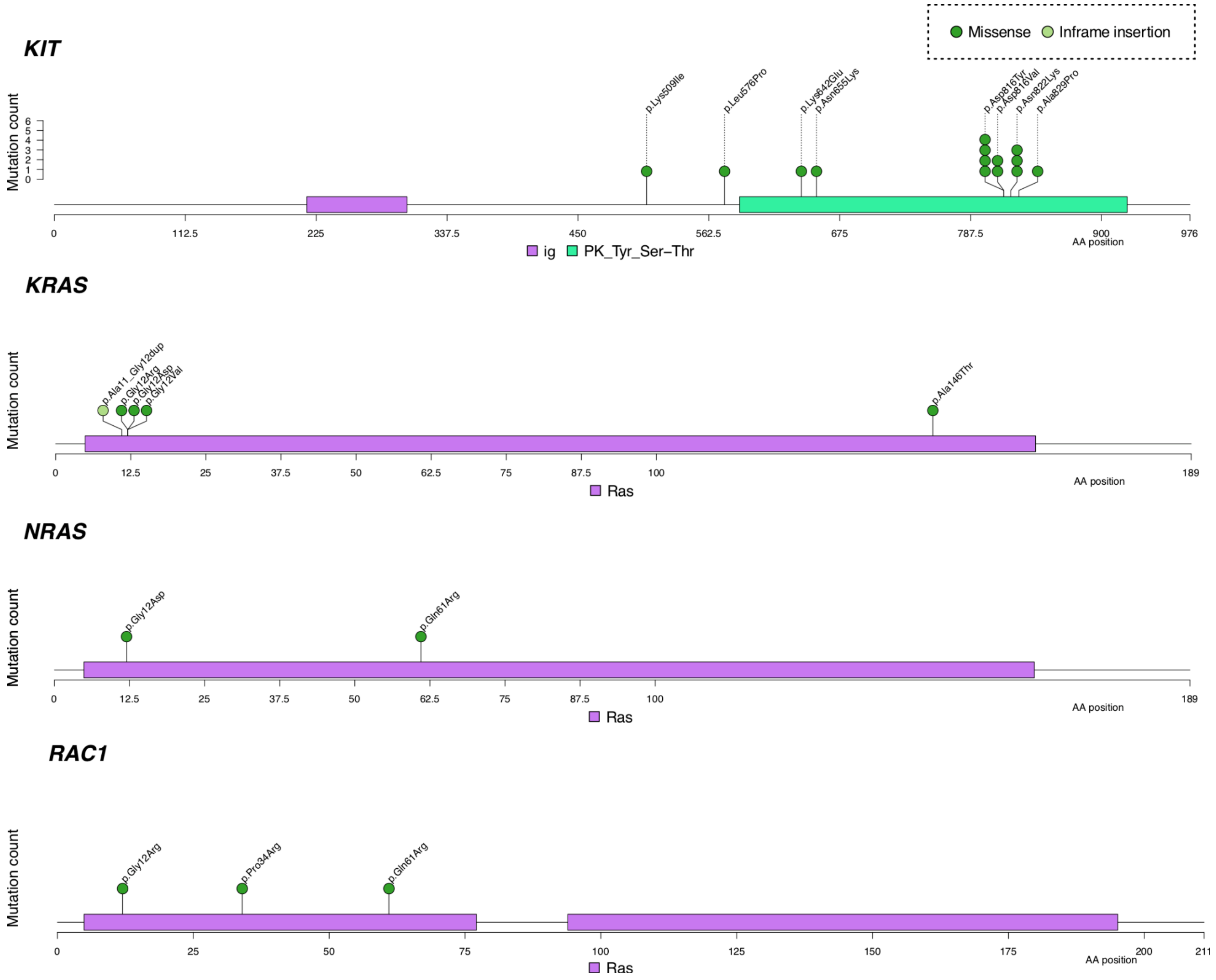

Supplementary Figure 2. Lolliplots showing the sites of putative driver variants in the GEL TGCT dataset in *KIT*, *KRAS*, *NRAS*, and *RAC1* as identified by the IntOGen pipeline. The color of the circles indicates variant type. Protein domains are from UniProt.

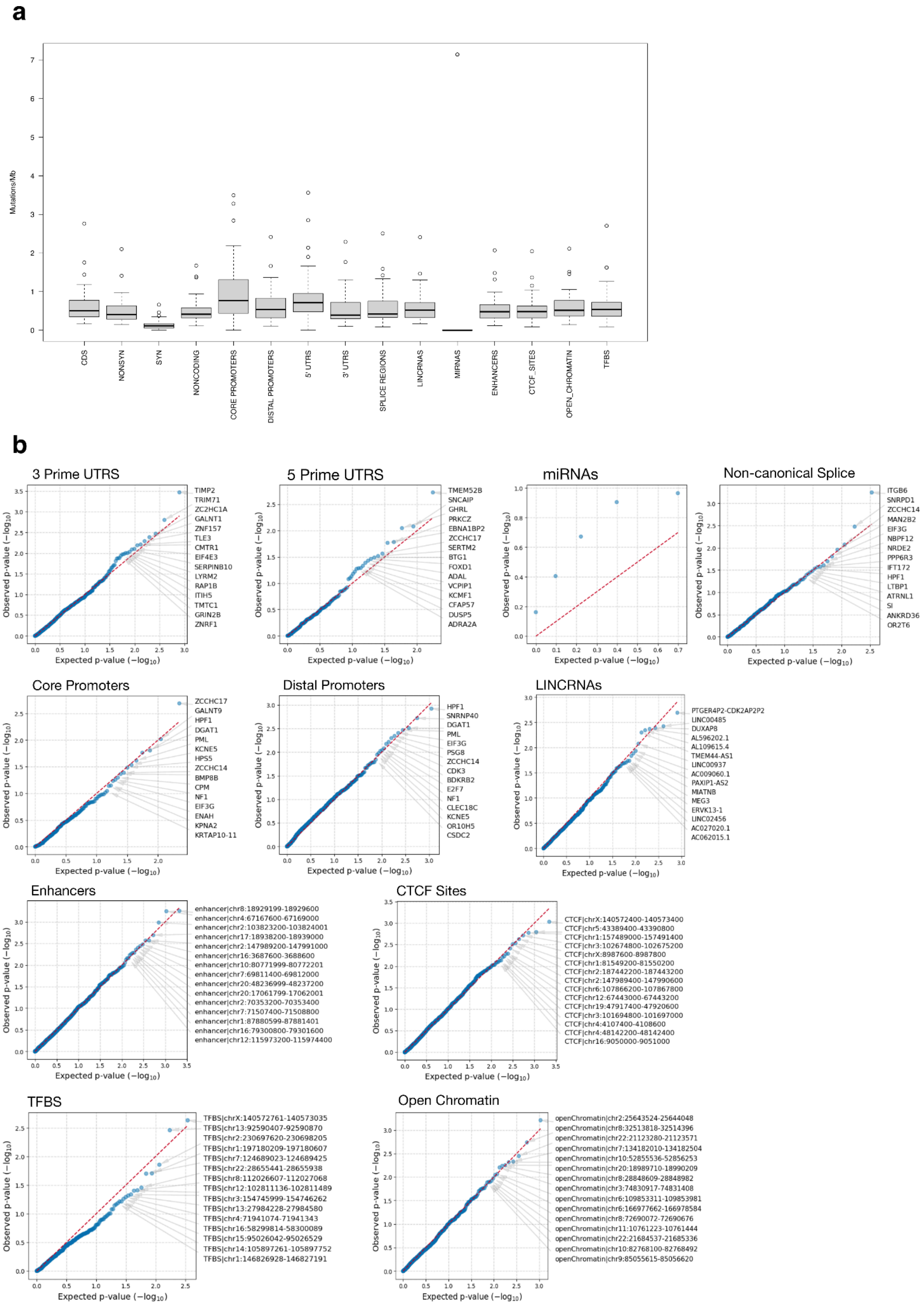

Supplementary Figure 3. **a.** Number of mutations per megabase (y-axis) for the following groupings (*left to right*): in CDS regions, non-synonymous, synonymous, non-coding, in core promoters, distal promoters, 5’ untranslated reading frames (UTRs), 3′ UTRs, splice regions,

long non-coding RNAs (LINCRNAS), microRNAs (MIRNAS), enhancers, CCCTC-binding factor (CTCF) sites, open chromatin, and transcription factor binding sites (TFBS) **b.** OncodriveFML results (*left to right, top to bottom*) as applied to identify putative non-coding drivers under positive selection in 3’ UTRs, 5′ UTRs, MIRNAs, non-canonical splice genes, core promoters, distal promoters, LINCRNAS, enhancers, CTCF sites, TFBS and regions of open chromatin. Centre line of boxplot: median, edges of box: first and third quartiles and the whiskers extend to the minimum and maximum values, excluding outliers.

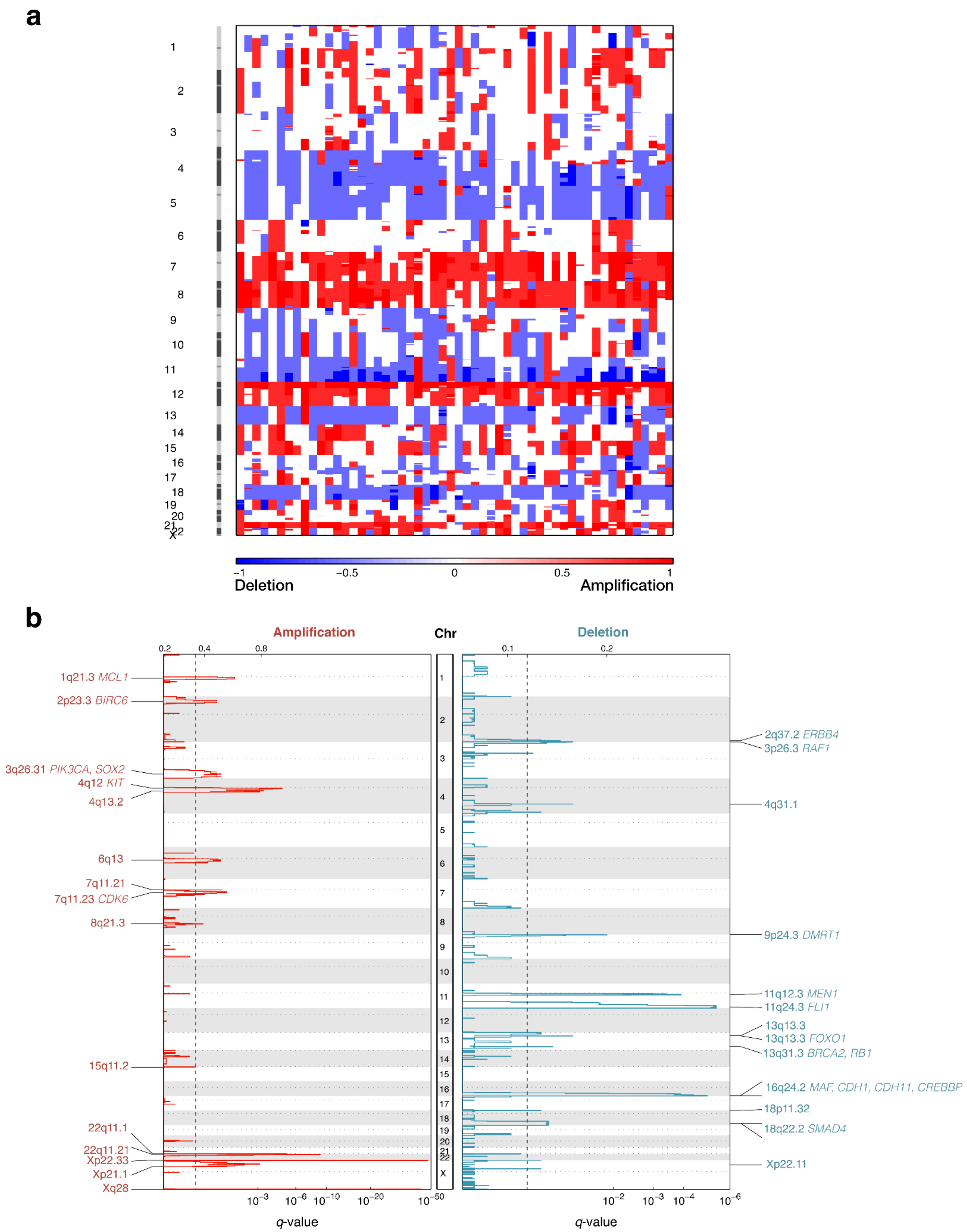

Supplementary Figure 4. a. Heatmap shows total segmented copy-number profiles across the cohort based on input copy number data. Chromosomes are arranged vertically from top to bottom and samples (unlabelled) are arranged horizontally from left to right. Red and blue

represent gain and loss, respectively. **b.** Regions of significant focal amplification (left) and deletion (right) identified with GISTIC2.0. Each plot shows G-scores (top) and q-values (bottom) with respect to amplifications and deletions over the entire region analyzed. Chromosome number is shown along the vertical axis. Dashed vertical line (q = 0.1) corresponds to the q-value cutoff by which significant targets or events were identified (see **Methods**). Focal events are labelled with cytoband names. Some of the identified potential cancer driver and predisposition genes are labelled at corresponding peaks (see **Supplementary Data 3**).

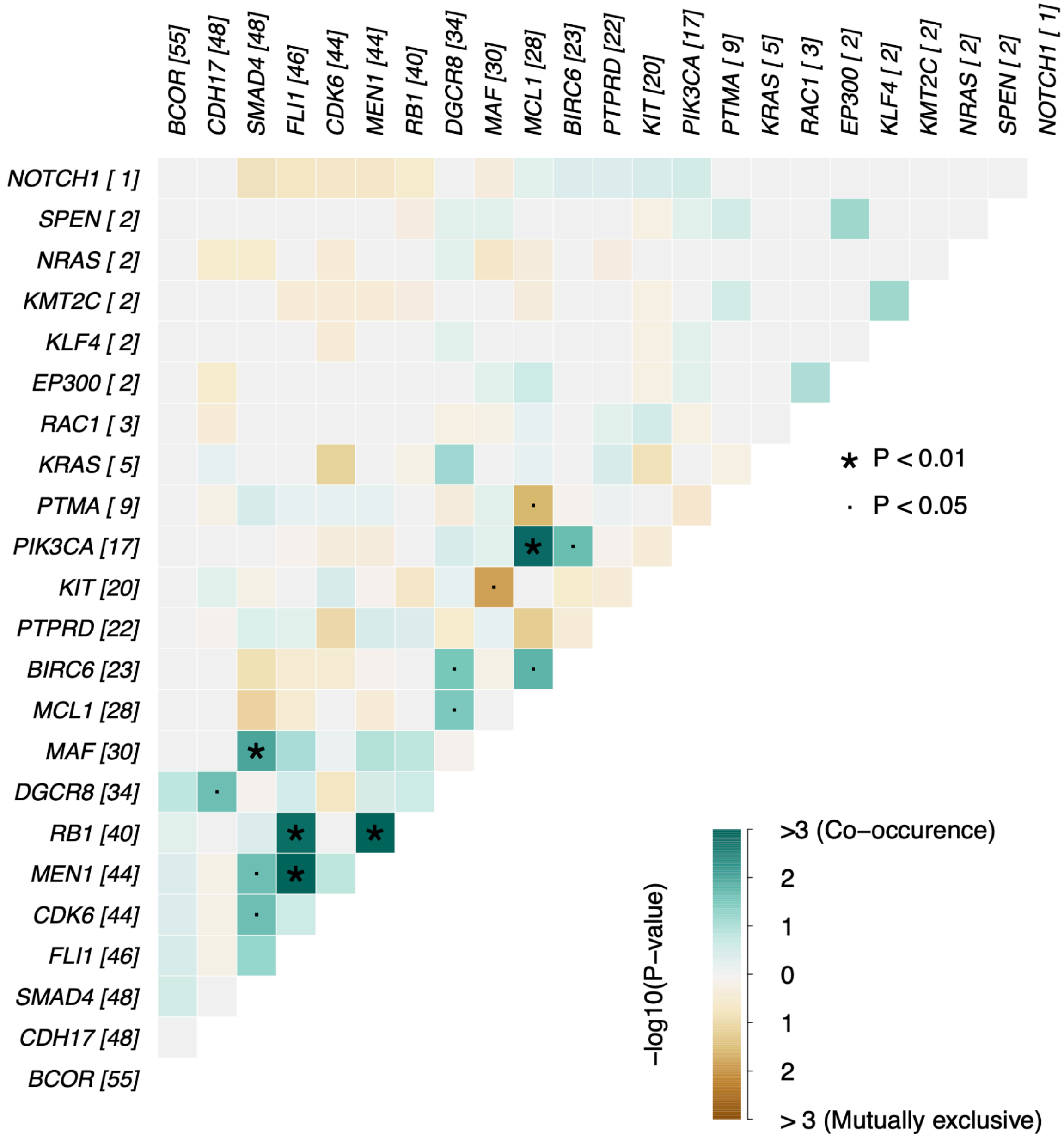

Supplementary Figure 5. Co-occurrence (green) and mutual exclusiveness (gold) between IntOGen-identified mutation-driver genes and GISTIC2-identified CNA-driver genes annotated with known oncogenes or tumour suppressor genes in the GEL TGCT dataset, determined using the R package maftools. Data pertains to 57 individual TGCT samples analysed (only one multi-region sample included).

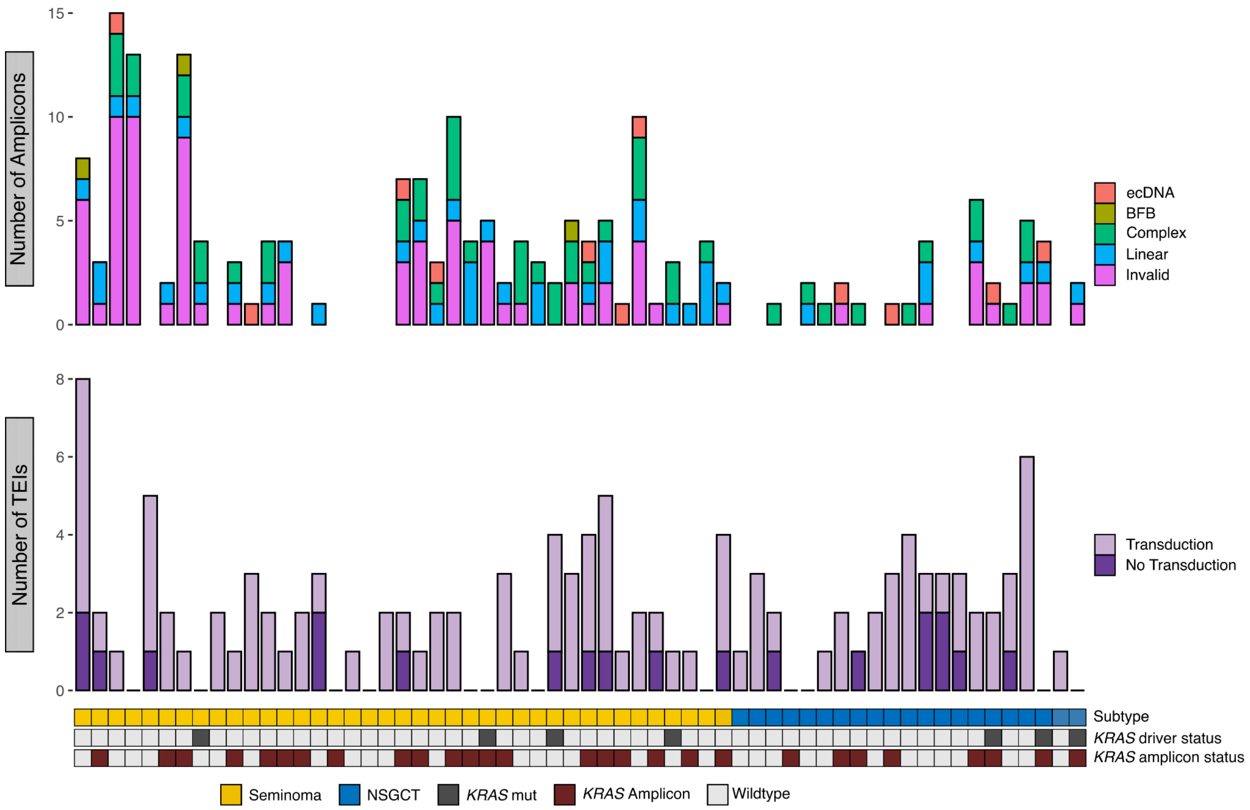

Supplementary Figure 6. From top to bottom: detailed overview of amplicon structures in GEL cohort, where amplicons are classified into extrachromosomal DNA (ecDNA) or circular, complex, linear, breakage-fusion-bridge (BFB), and invalid; number of transposable element insertions (TEIs) across all samples; TGCT disease subtype; mutation status of *KRAS* driver gene; presence or absence of amplicon structure involving *KRAS*.

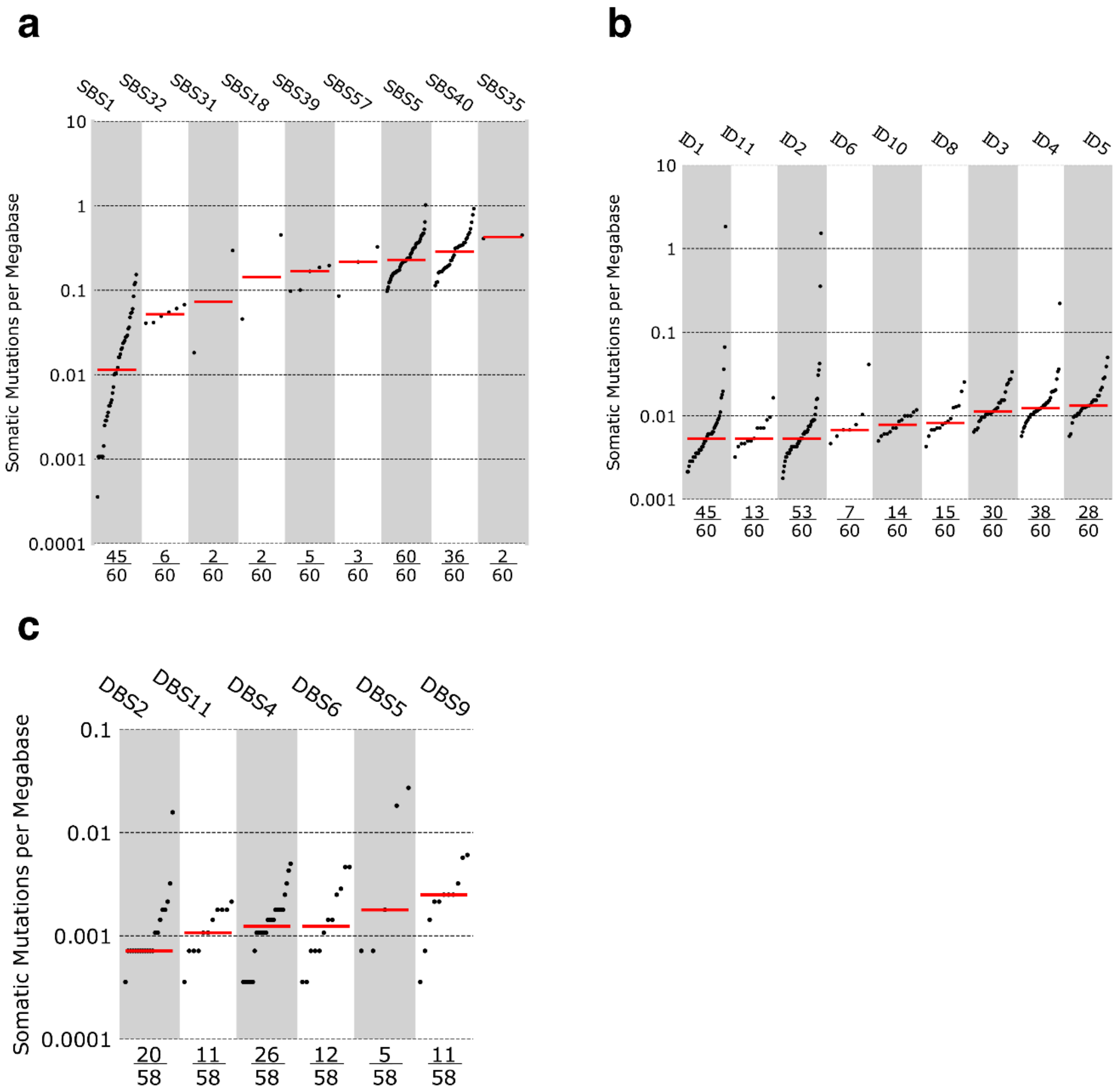

Supplementary Figure 7. a. SBS, **b**. ID and **c.** DBS mutational signature analysis. Each black dot represents a sample in the GEL TGCT dataset while the red horizontal lines are the median numbers of mutations in the respective signature types. The vertical axis shows the number of somatic mutations per megabase and the different signature exposures are ordered on the horizontal axis based on their median numbers of somatic mutations.

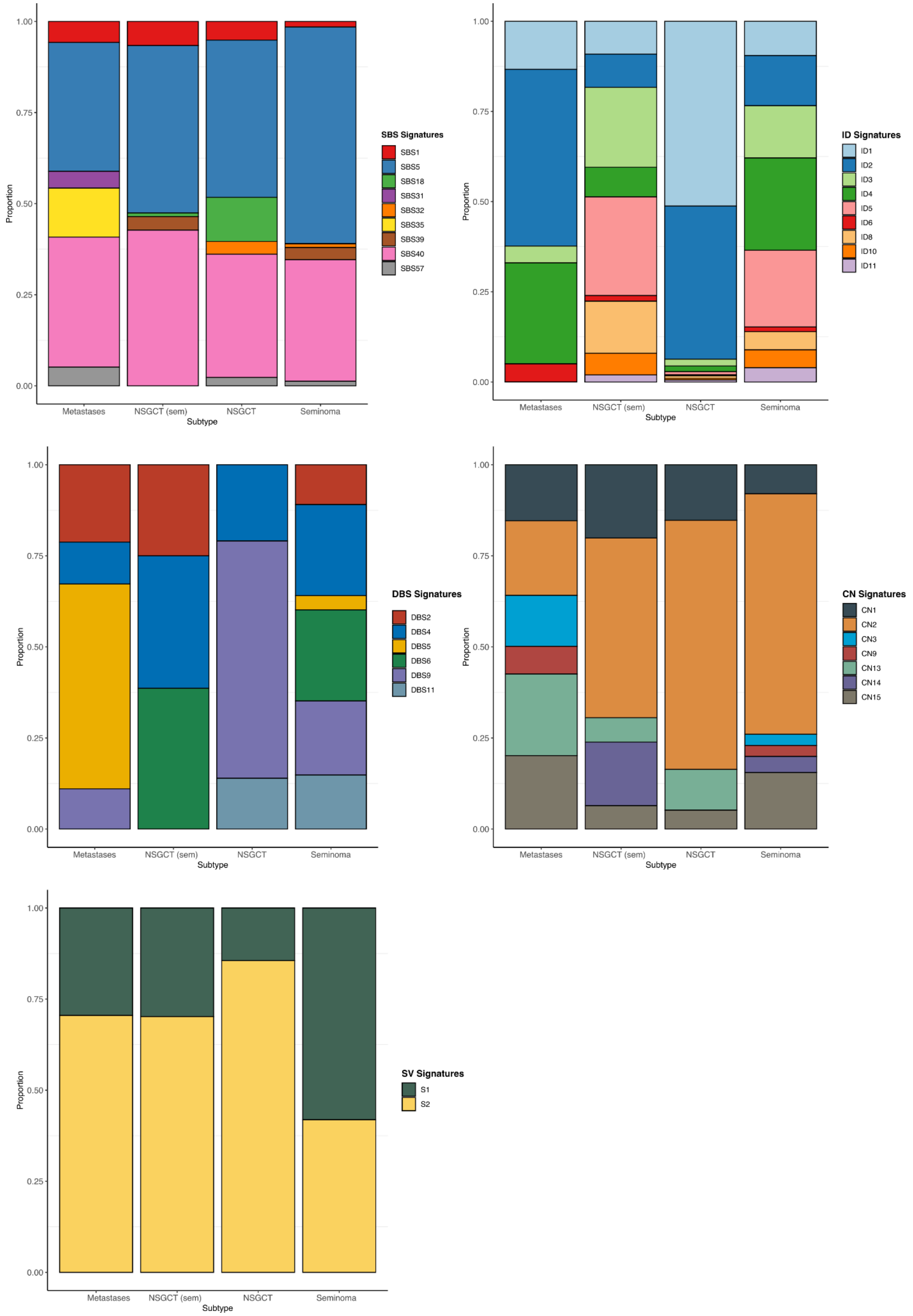

Supplementary Figure 8. Mutational spectra and signatures in testicular germ cell

tumours (TGCT) Proportion of mutations attributed to single base substitutions (SBS; *top left)*, small insertion and deletions (ID; *top right*), doublet base substitutions (DBS; *middle left*), copy number event (CN; *middle right*) and structural variant (SV; *bottom left*) signatures across all 60 TGCTs and according to subtype. NSGCT, non-seminomatous germ cell tumour excluding those with seminomatous histological components; NSGCT (sem), non-seminomatous germ cell tumours *including* those with seminomatous histological components.

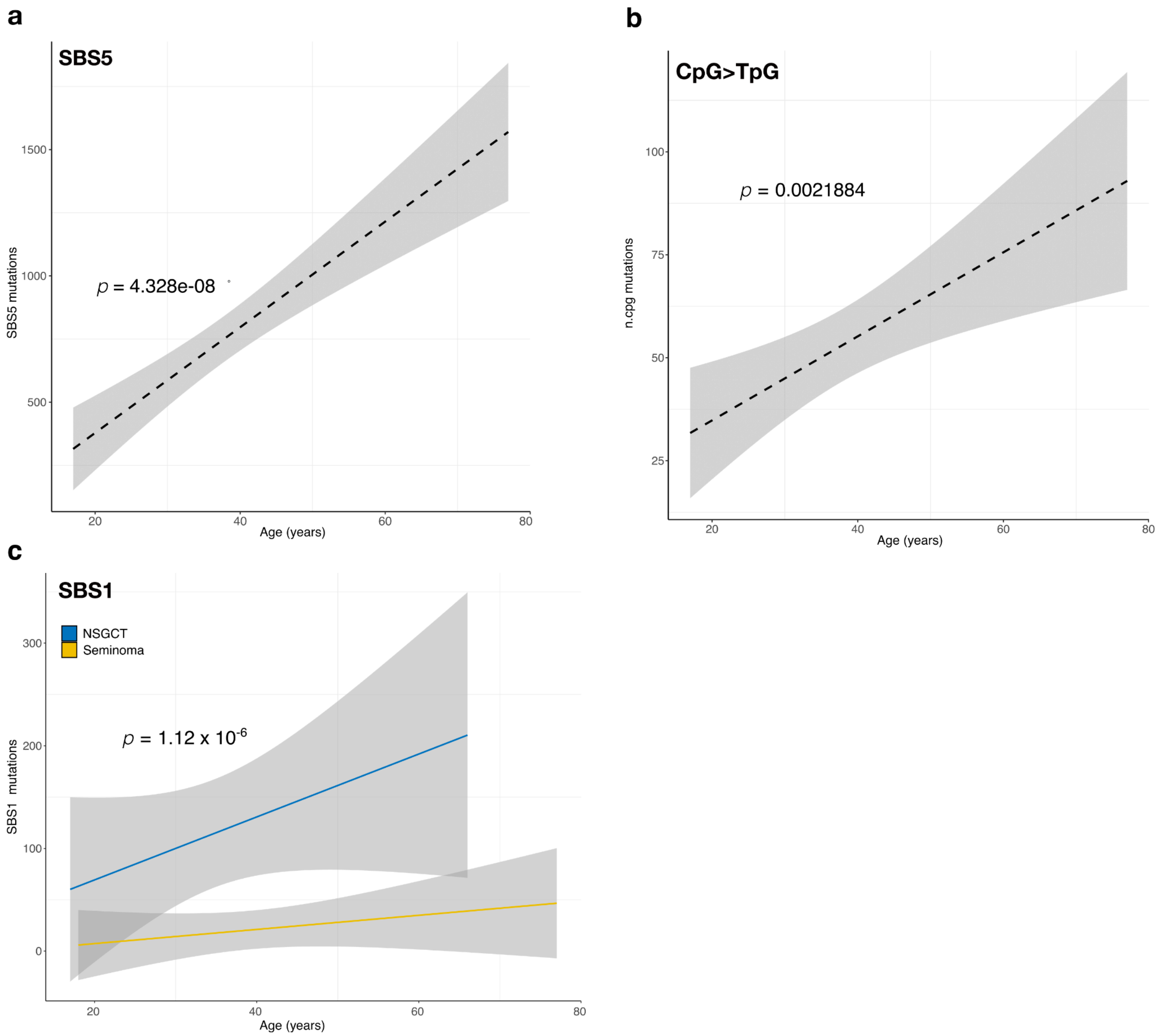

Supplementary Figure 9. a. Correlations between age of cancer diagnosis and mutations attributed to signature 5 (SBS5) and **b.** C>T substitutions at CpG sites (CpG>TpG). The y-axes correspond to the numbers of somatic substitutions attributed to either signature, while x-axes correspond to the ages of diagnosis. Dashed lines show best estimates for the slopes derived from a robust linear regression. 95% confidence intervals for the slopes are shown in light grey shading. **c.** Correlations between age of cancer diagnosis and mutations attributed to signature 1 (SBS1) split by histological subtype. P-value shown on is from a two-sided Wilcoxon rank sum test comparing SBS1 mutation burden between seminomas and NSGCT. NSGCT, Non-seminomatous germ cell tumour.

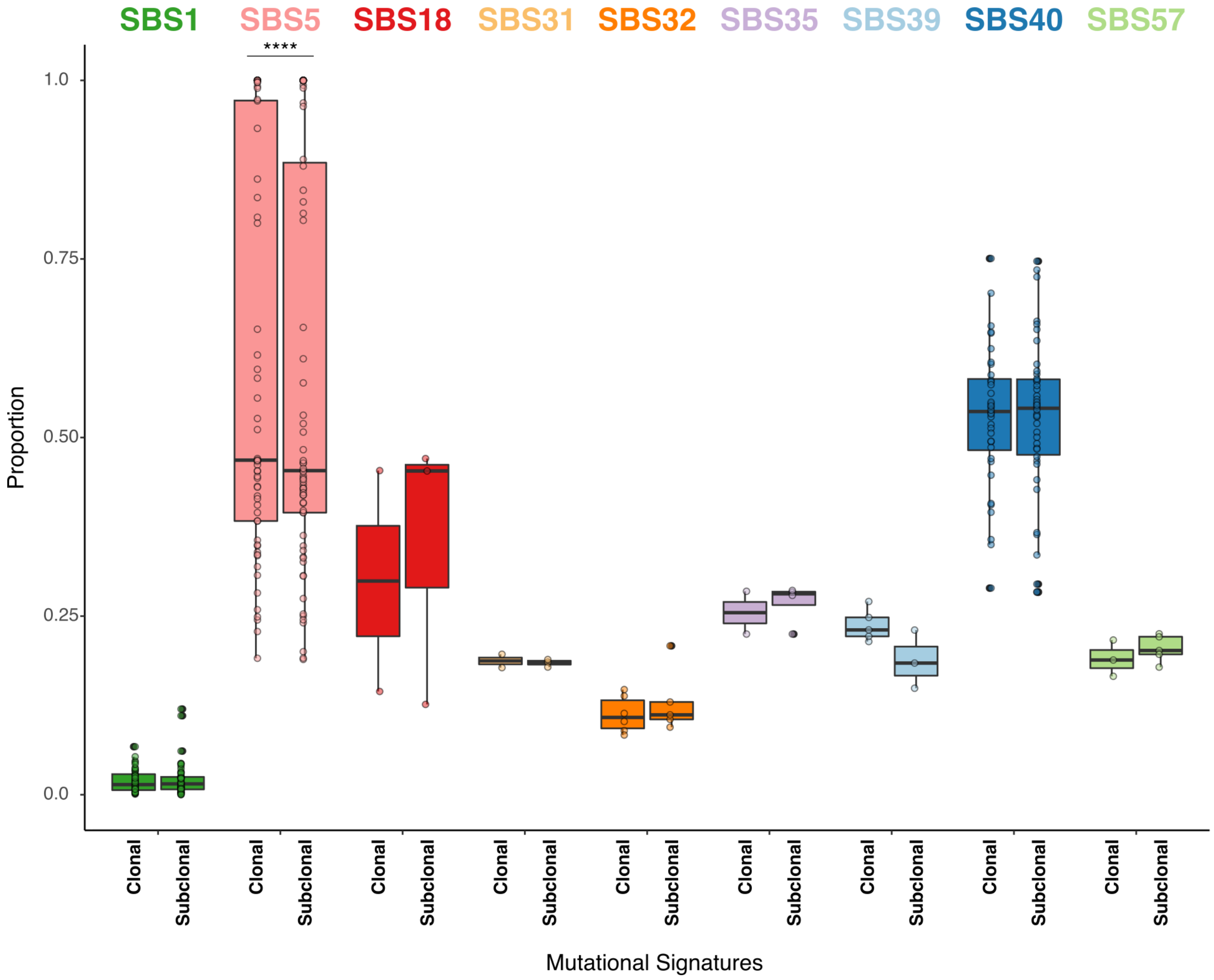

Supplementary Figure 10. Proportion of clonal and subclonal mutations attributed to each COSMIC single base substitution (SBS) signature across the GEL TGCT cohort. **** indicates a p value ≤ 0.0001. Centre line of boxplot: median, edges of box: first and third quartiles and the whiskers extend to the minimum and maximum values, excluding outliers.

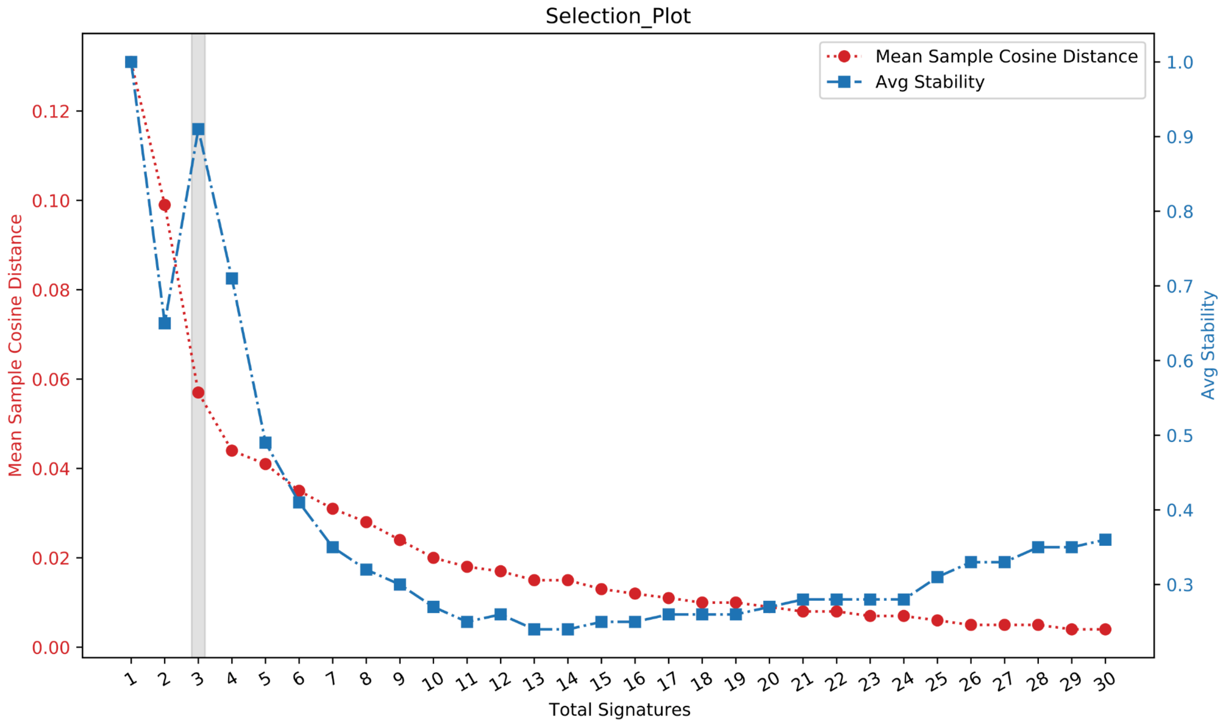

Supplementary Figure 11. Copy Number Signature Selection Plot. Grey bar indicates the optimum number of de novo signatures identified in the GEL TGCT dataset.

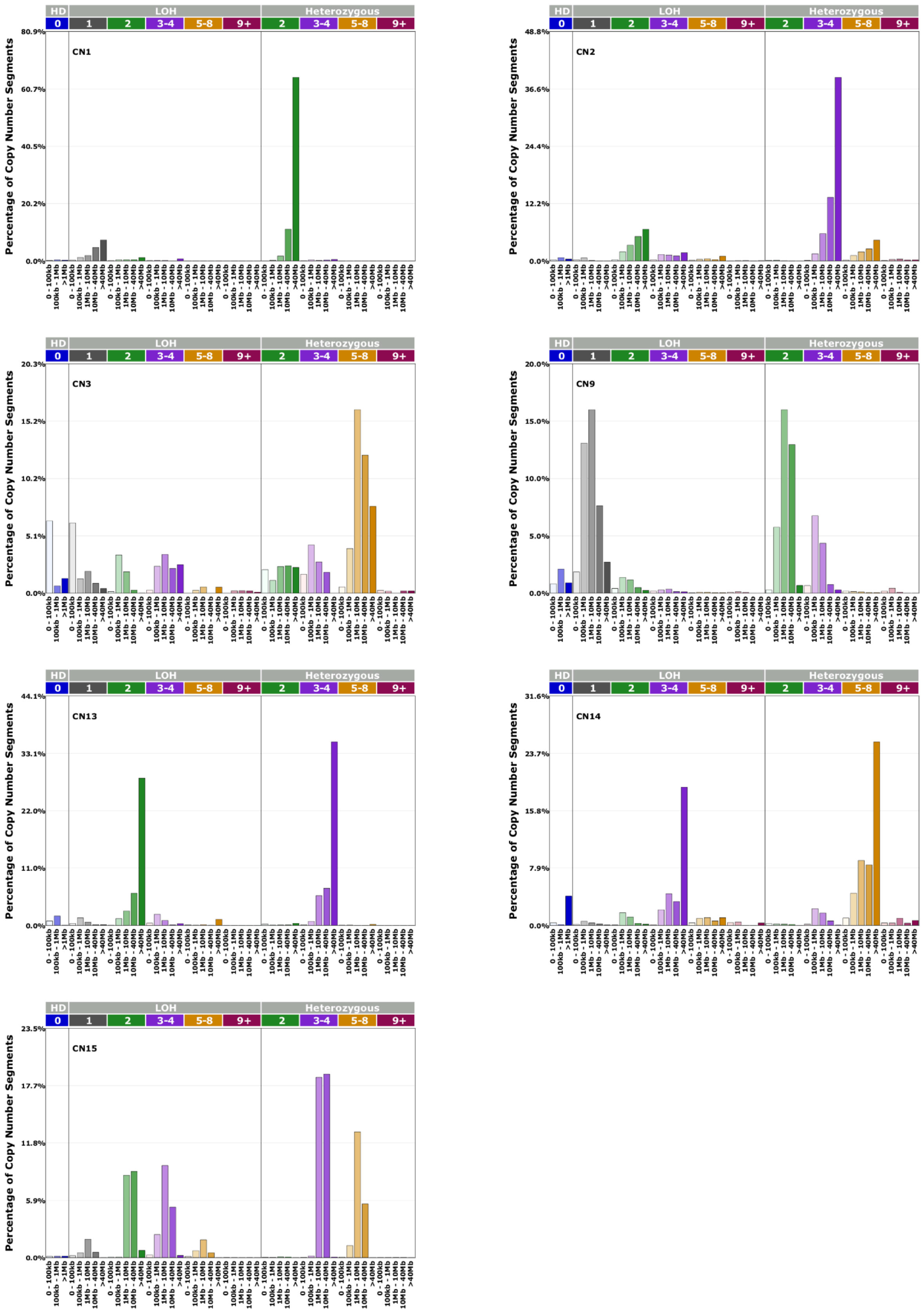

Supplementary Figure 12. Decomposition plots for 7 COSMIC copy number signatures (CN1,

CN2, CN3, CN9, CN13, CN14, CN15) identified in the GEL TGCT dataset. Heterozygosity status and total copy number (0–9+) are indicated above each column. Segment sizes are indicated below each column. Increasing saturation of colour indicates increasing segment size. LOH, loss of heterozygosity; HD, homozygous deletion.

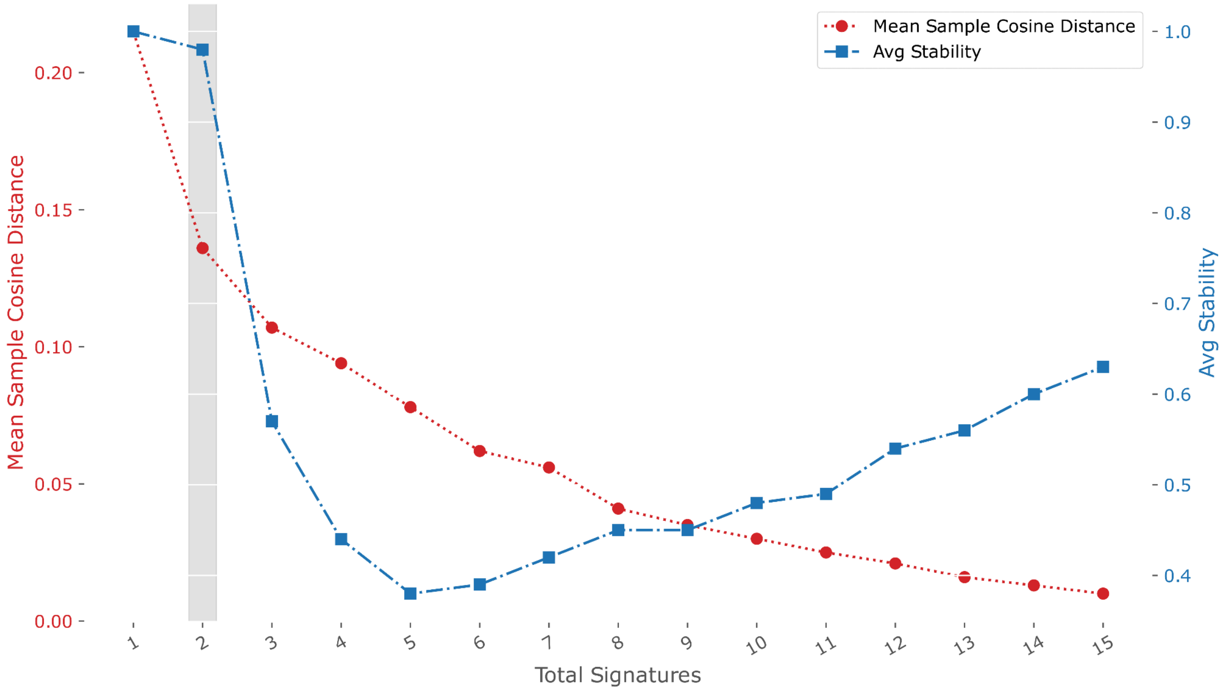

Supplementary Figure 13. Structural Variant Signature Selection Plot. Grey bar indicates the optimum number of de novo signatures identified in the GEL TGCT cohort.

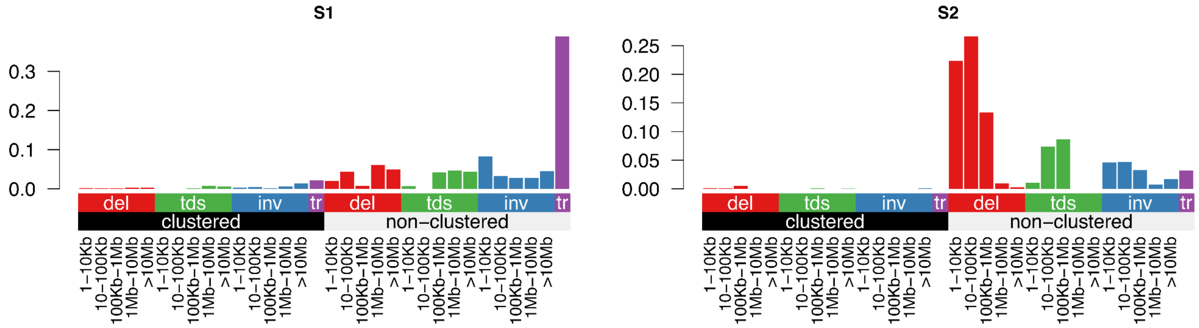

Supplementary Figure 14. Structural variant signatures S1 and S2 extracted de novo from GEL TGCT dataset using non-negative matrix factorization. Probability of rearrangement element shown on y-axis. Rearrangement elements are categorized by type and sorted by size within clustered and non-clustered groups along the x-axis. del, deletion; tds, tandem duplication; inv, inversion; tr, translocation.

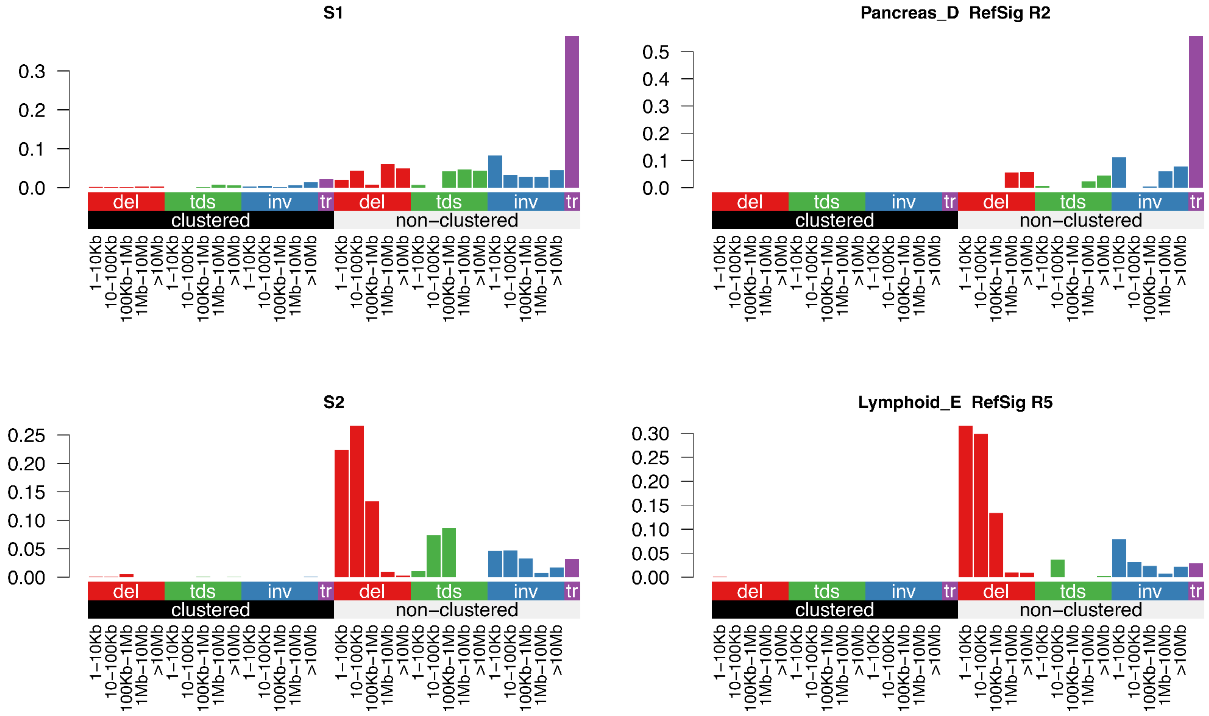

Supplementary Figure 15. Comparison of GEL SV signatures with (S1, S2) signatures extracted using the PCAWG study (RefSig R2, RefSig R5). Signatures extracted de novo from the current study are shown on the left while the most similar organ specific PCAWG signature is presented on the right. Probability of rearrangement element shown on y-axis. Rearrangement elements are categorized by type and sorted by size within clustered and non-clustered groups along the x-axis. del, deletion; tds, tandem duplication; inv, inversion; trans, translocation.

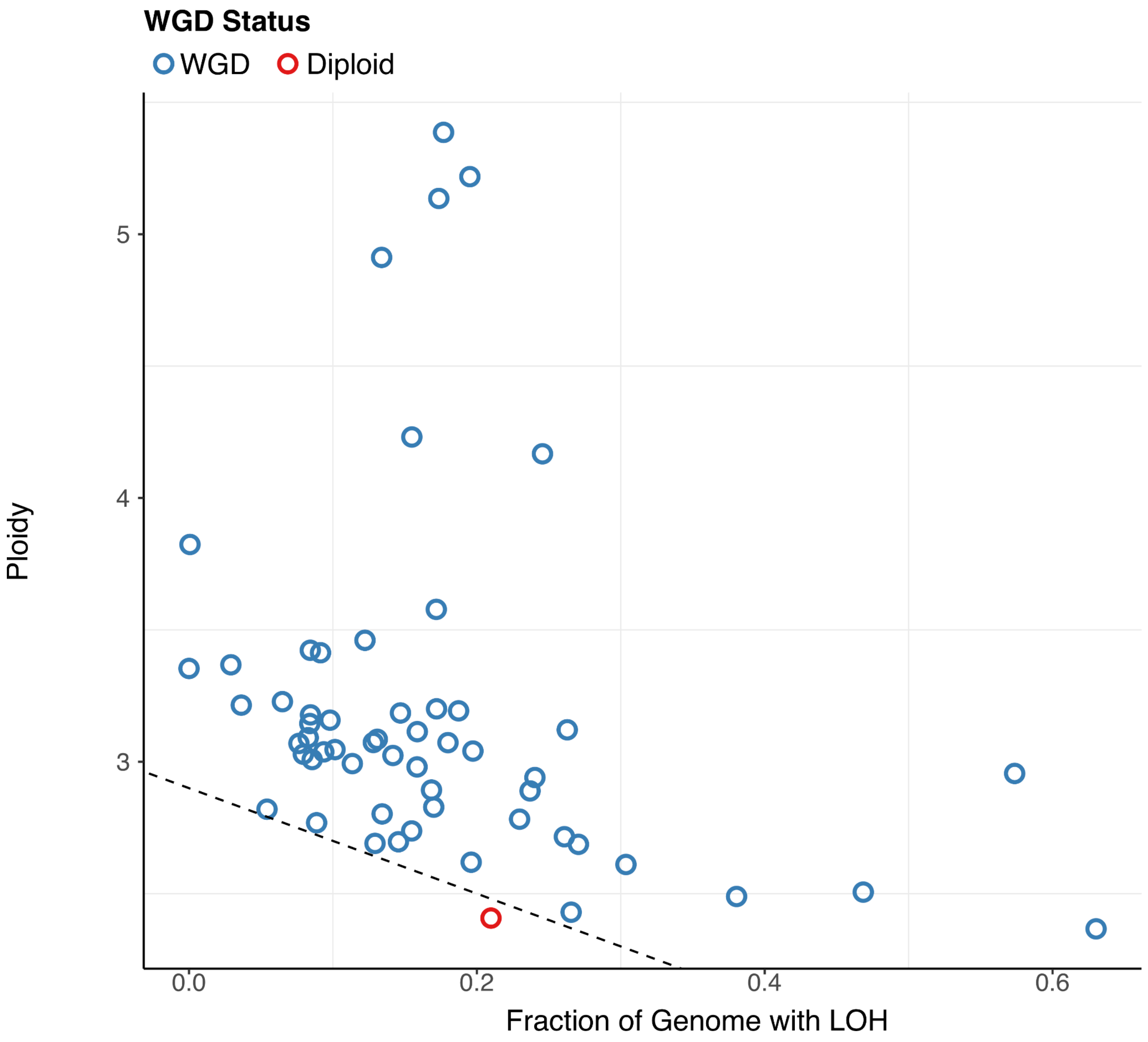

Supplementary Figure 16. Classification of GEL TGCT samples that had undergone whole-genome duplication (WGD) based on ploidy and the fraction of the genome displaying loss of heterozygosity (LOH). Samples were determined to have undergone whole genome duplication when their ploidy (weighted by subclonality) was ≥ 2.9 – 2 * fraction of genome with LOH (weighted by subclonality). This approach was established and validated by the PCAWG Consortium.

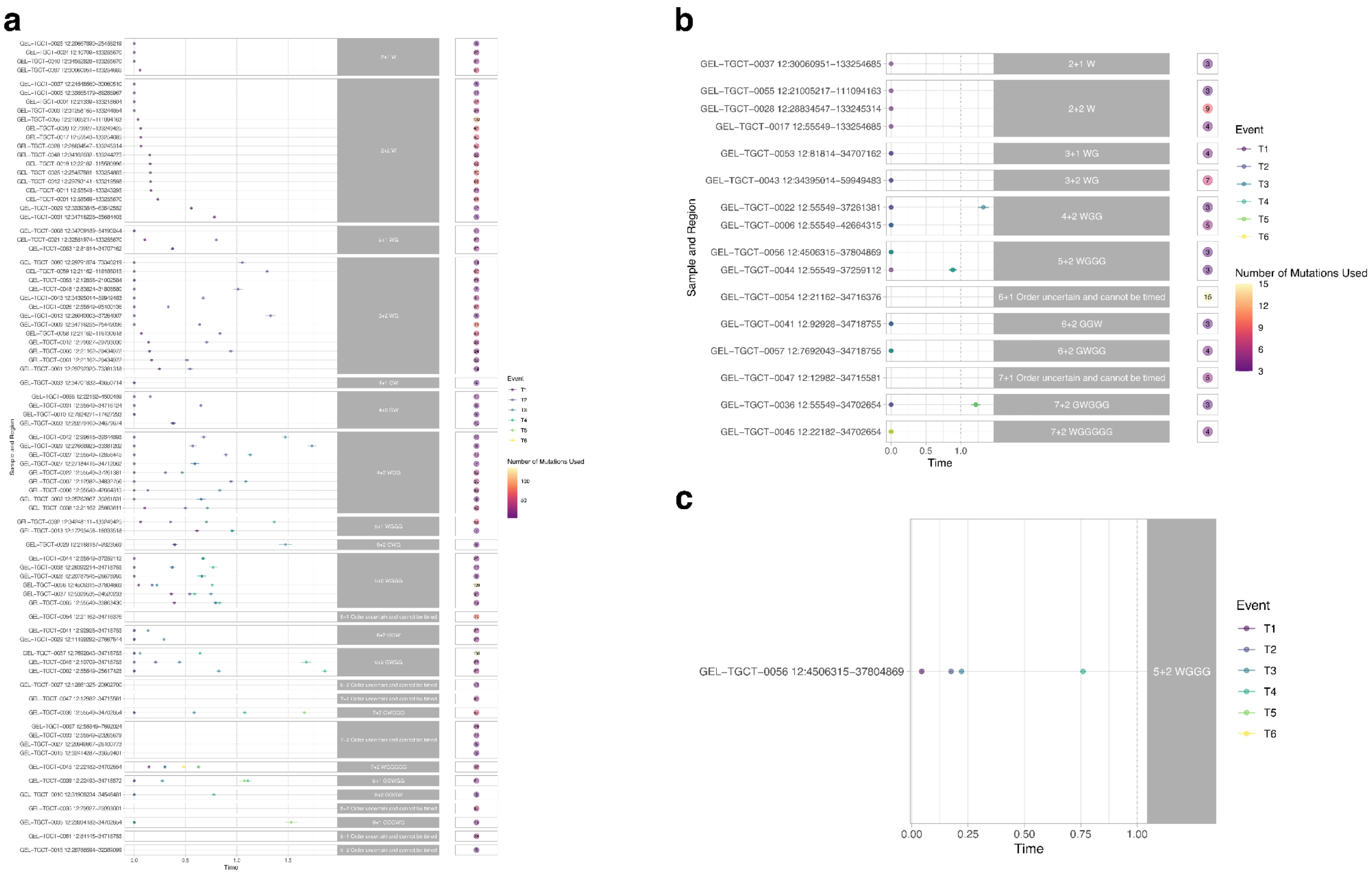

Supplementary Figure 17. AmplificationTimeR results inferring the order of high-level focal gains (G) on chromosome 12 relative to whole genome duplication (W). The sequence of G and W indicates the order of events that led to the final copy number state, e.g. 5+2 GWG indicates that a final copy number state of 5+2 was reached through a single gain event followed by a whole genome duplication and a final gain event occurring after the whole genome duplication. Horizontal coloured bars span the 95% confidence intervals. Individual dots correspond to individual gains of the same segment averaged across 500 bootstrapped samples. **a.** 79 regions timed across 39 individual samples using all mutations. Each sample is plotted individually and information is not shared between segments from the same individual. Samples are grouped by the final copy number state and order of events. **b.** Gained segments timed using only clocklike C>T at CpG mutations. 16 regions originating from 13 samples were timed. **c.** Example where a sample (GEL-TGCT-0056) has many mutations. All of the possible multiplicity states for this sample are covered resulting in a high-confidence ordering of events.

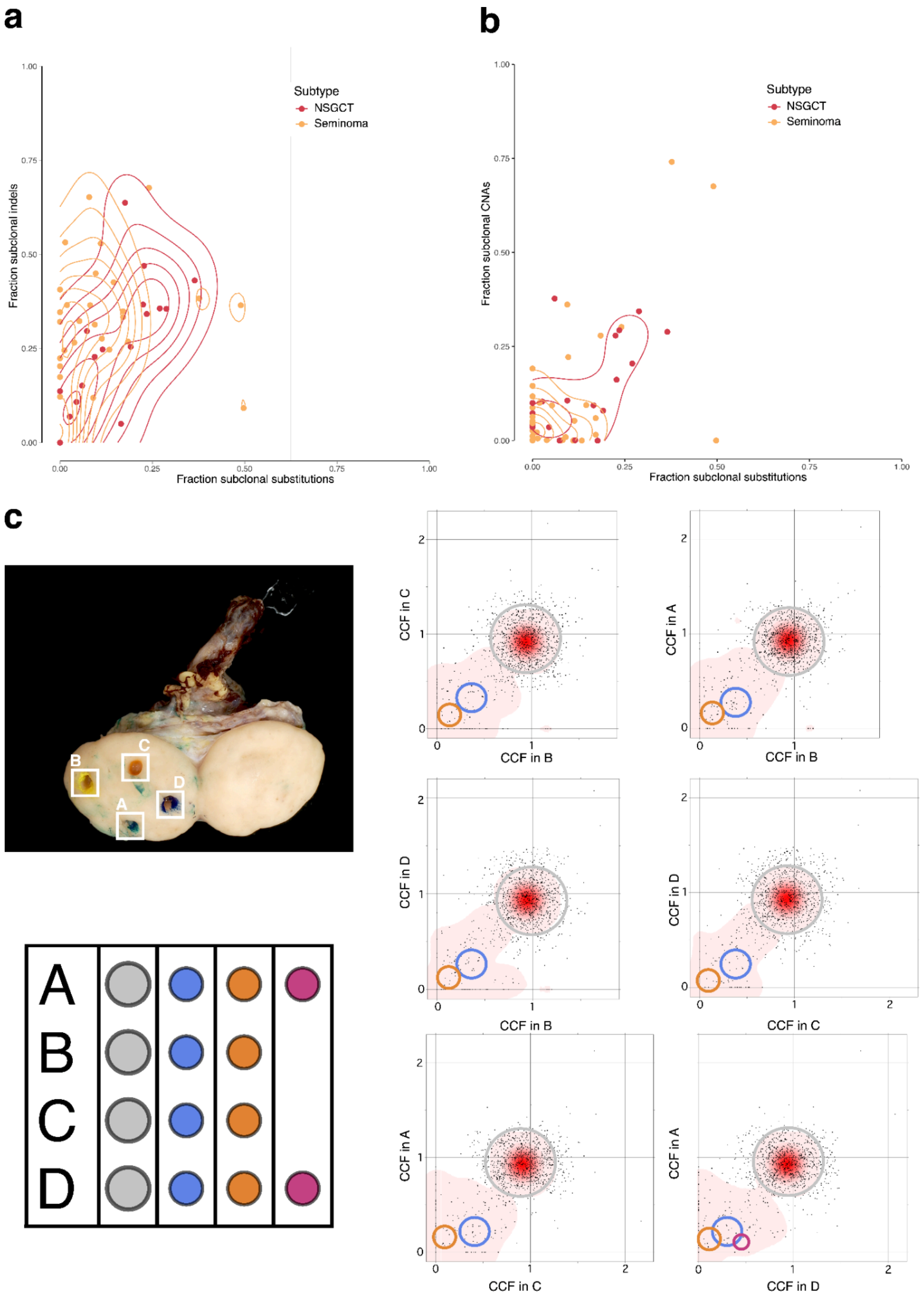

Supplementary Figure 18. a. The x-axis shows the fraction of SNVs that are subclonal and the y-axis the fraction of indels that are subclonal. **b.** The x-axis shows the fraction of SNVs that are subclonal and the y-axis the fraction of copy number alterations that are subclonal. Each dot represents a different sample, colored by subtype. Contour lines calculated using MASS::kde2d() in R ; n = 57 biologically independent samples. **c.** Multidimensional Bayesian Dirichlet Process-based mutation clustering (ndDPClust) was used to identify truncal, clonal and subclonal mutation clusters across 4 tumour regions (A, B, C, D) sampled from a primary pure seminoma in participant GEL-TGCT-0058. The subclonal composition of individual samples is

shown (*left*) where colors correspond to distinct clusters. Cancer Cell Fractions (CCF) – denoting the proportion of cancer cells carrying a mutation within a sample – are shown for each pair (*right*) and for all substitutions detected. Areas of dense red shading, positioned away from the axes and with CCF >0 and <1, indicate the presence of mutation clusters at subclonal levels across multiple sites.

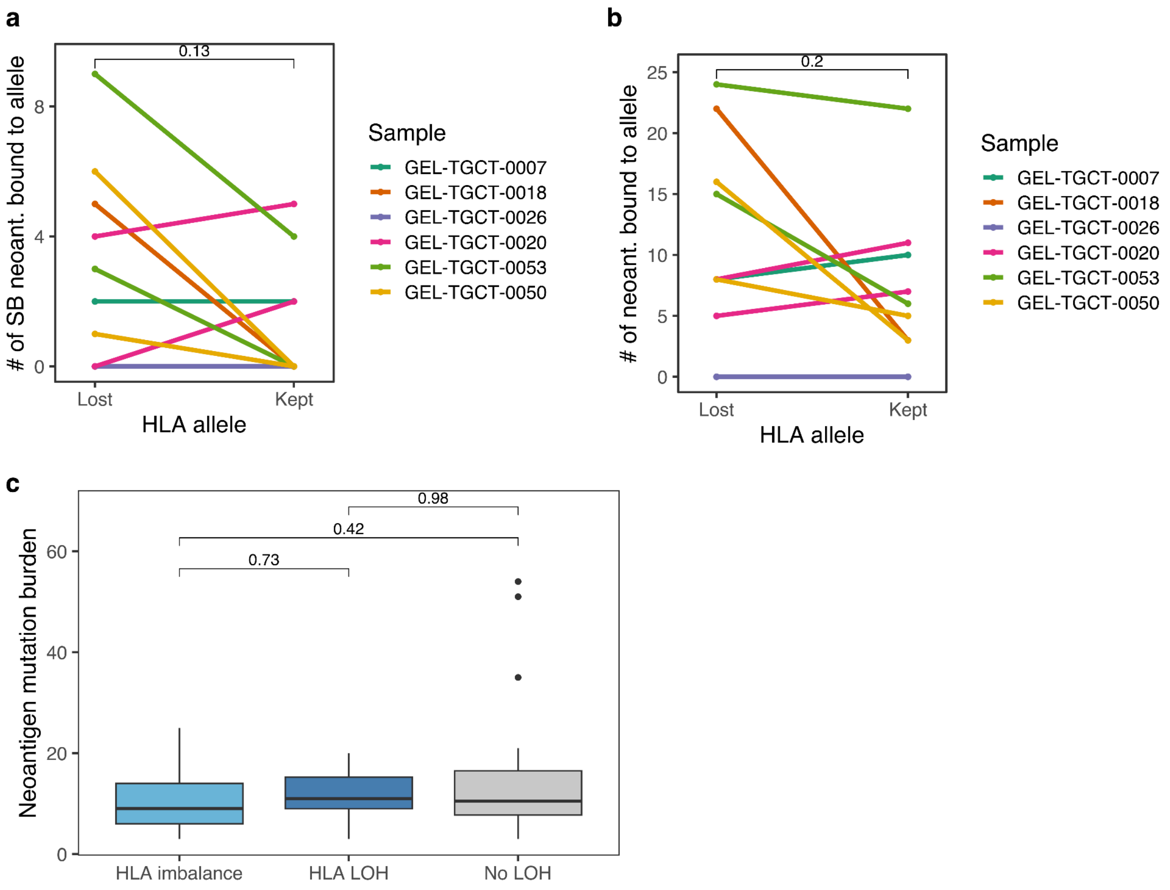

Supplementary Figure 19. a. Counts showing number of strong MHC-binding (SB) neoantigens with affinity to lost (left) and to kept (right) alleles in six GEL samples **b.** Counts showing number of MHC-binding neoantigens with affinity to lost (left) and to kept (right) allele in six GEL samples **c.** Estimated neoantigen burden in HLA imbalance (left), HLA LOH (centre) and No LOH (right). Values connecting sample groups indicate the p-values of paired Wilcoxon signed-rank tests (a, b) and Wilcoxon rank-sum test (c). Centre line of boxplot: median, edges of box: first and third quartiles and the whiskers extend to the minimum and maximum values, excluding outliers.

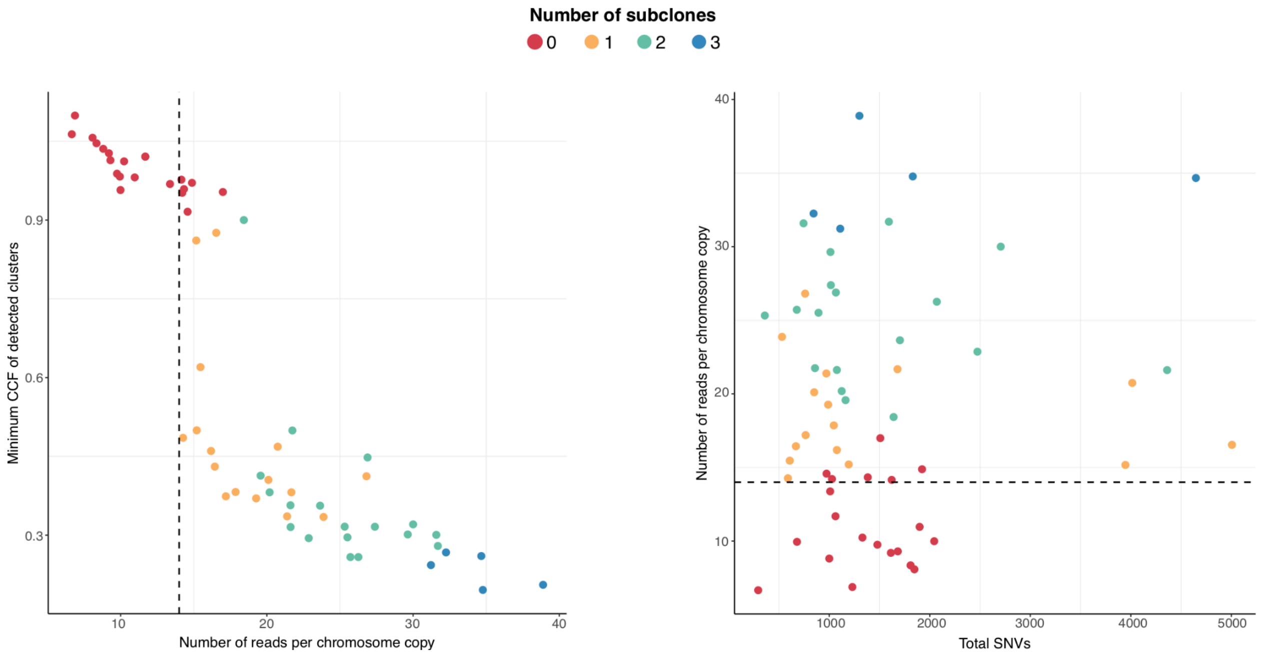

Supplementary Figure 20. Minimum cancer cell fraction (CCF) of the DPClust detected clusters plotted against the number of reads per chromosome copy (nrpcc) in each tumour (*left panel*). Nrpcc versus the total number of SNVs in each tumour (*right panel*). Data point colors correspond to the total number of DPClust clusters detected per tumour. Downstream analysis of mutation timing is limited to with a minimum nrpcc of 14 (black dashed line).

Genomic landscape of adult testicular germ cell tumours in the 100,000 Genomes Project

Supplementary Note 1

Consortia members

The numbering of affiliations within consortia follows the convention that affiliations already listed in the main author list retain their original number.

Testicular Genomics England Clinical Interpretation Partnership Consortium (GECIP)

Richard Y. Ball[13], Ksenija Benes[15], Daniel M. Berney[23], Lisa Browning[24,25], Maite Cabes[26], Daniel Chubb[5], David Church[25,27], Nicholas Coleman[20,28], Alex J. Cornish[5], Benjamin P. Fairfax[29], Anna Frangou[4], Robert Huddart[30], Johnathan K. Joffe[31], Ben Kinnersley[5,6], Sally Lane[32], Alexander T. J. Lee[12], Cathryn Leng[33], Kevin Litchfield[34], Danish Mazhar[35], Iain A. McNeish[36], Rhodri Morgans[37], Matthew J. Murray[19,20], Máire Ní Leathlobhair[1,2,3], Thomas Powles[23], Andrew Protheroe[18], Jane Rogan[12], Jonathan Shamash[38], Janet Shipley[39], Rushan Sylva[14], Sara Stoneham[40], Jenny C. Taylor[25,27], Ian P. M. Tomlinson[29], Clare Turnbull[5,30], Dan Stark[16], Christopher S. Sweeney[41], Eliezer M. Van Allen[41], Clare Verrill[21,22], David C. Wedge[1,11,12], Robin J. Young[17]

1. Big Data Institute, Nuffield Department of Medicine, University of Oxford, Oxford, UK
2. Ludwig Institute for Cancer Research, Nuffield Department of Medicine, University of Oxford, Oxford, UK
3. Department of Microbiology, Moyne Institute of Preventive Medicine, School of Genetics and Microbiology, Trinity College Dublin, Ireland
4. Max Planck Institute of Molecular Cell Biology and Genetics, Dresden, Germany
5. Division of Genetics and Epidemiology, The Institute of Cancer Research, London, UK
6. University College London Cancer Institute, 72 Huntley Street, London, UK
7. Department of Mathematical Sciences, Chalmers University of Technology and University of Gothenburg, Gothenburg, Sweden
8. Genomics England, London, UK
9. Department of Biology, University of Konstanz, Universitaetsstrasse 10, D-78464 Konstanz, Germany
10. Manchester Cancer Research Centre, The University of Manchester, Manchester, UK
11. Division of Cancer Sciences, University of Manchester, Manchester Academic Health Science Centre, Manchester, UK
12. Christie Hospital, The Christie NHS Foundation Trust, Manchester Academic Health Science Centre, Manchester, UK
13. Norfolk and Norwich University Hospitals NHS Foundation Trust, Norwich, UK
14. Guy's and St Thomas' NHS Foundation Trust, London, UK
15. Department of Pathology, The Royal Wolverhampton NHS Trust, UK
16. Leeds Institute of Medical Research at St James's, University of Leeds, Leeds, UK
17. Weston Park Cancer Centre, Sheffield Teaching Hospitals NHS Foundation Trust, Sheffield, UK
18. Department of Oncology, Oxford University Hospitals NHS Foundation Trust, Oxford, UK

19. Department of Paediatric Haematology and Oncology, Cambridge University Hospitals NHS Foundation Trust, Cambridge, UK
20. Department of Pathology, University of Cambridge, Cambridge, UK
21. NIHR Oxford Biomedical Research Centre, Oxford, UK
22. Nuffield Department of Surgical Sciences, University of Oxford, Oxford, UK
23. Barts Cancer Institute, Queen Mary University of London, London, UK
24. Department of Cellular Pathology, Oxford University Hospitals NHS Foundation Trust, Oxford, UK
25. NIHR Oxford Biomedical Research Centre, John Radcliffe Hospital, Oxford University Hospitals NHS Foundation Trust, Oxford, UK
26. Oxford Molecular Diagnostics Centre, John Radcliffe Hospital, Oxford University Hospitals NHS Foundation Trust, Oxford, UK
27. Wellcome Centre for Human Genetics, University of Oxford, Oxford, UK
28. Department of Histopathology, Addenbrooke's Hospital, Cambridge University Hospitals NHS Foundation Trust, Hills Road, Cambridge, UK
29. Department of Oncology, University of Oxford, Oxford, UK
30. Royal Marsden Hospital, London, UK
31. St James's University Hospital, Leeds, UK
32. Yorkshire and North East Genomic Medicine Service Alliance, Leeds, UK
33. Weston Park Cancer Centre, Sheffield Teaching Hospitals NHS Foundation Trust, Sheffield, UK
34. Cancer Research UK Lung Cancer Centre of Excellence, University College London Cancer Institute, London, UK
35. Department of Oncology, Addenbrooke's Hospital, Cambridge University Hospitals NHS Foundation Trust, Hills Road, Cambridge, UK
36. Department of Surgery and Cancer, Ovarian Cancer Action Research Centre, Imperial College London, London, UK
37. University Hospitals Birmingham NHS Foundation Trust, Birmingham, UK
38. Department of Medical Oncology, Barts Health NHS Trust, London, UK
39. Sarcoma Molecular Pathology, Division of Molecular Pathology, The Institute of Cancer Research, London, UK
40. Department of Paediatric Oncology, University College London Hospitals NHS Foundation Trust, London, UK
41. Dana-Farber Cancer Institute, Boston, MA, USA

Genomics England Research Consortium

Ambrose J. C.[8] , Baple E. L.[8] , Bleda M.[8] , Boardman-Pretty F.[8,42] , Boissiere J. M.[8] , Boustred C. R.[8], Caulfield M. J.[8,42], Chan G. C.[8], Craig C. E. H. [8], Daugherty L. C.[8], de Burca A.[8], Devereau, A.[8], Elgar G.[8,42], Foulger R. E.[8], Fowler T.[8], Furió-Tarí P.[8], Hackett J. M.[8], Halai D.[8], Holman J. E.[8], Hubbard T. J. P.[8], Jackson R.[8], Kasperaviciute D.[8,42], Kayikci M.[8], Lahnstein L.[8], Lawson K.[8], Leigh S. E. A.[8], Leong I. U. S.[8], Lopez F. J.[8], Maleady-Crowe F.[8], Mason J.[8], McDonagh E. M.[8,42], Moutsianas L.[8,42], Mueller M.[8,42], Murugaesu N.[8], Need A. C.[8,42], Odhams C. A.[8], Patch C.[8,42], Perez-Gil D.[8], Polychronopoulos D.[8], Pullinger J.[8] , Rahim T.[8], Rendon A.[8] , Riesgo-Ferreiro P.[8], Rogers T. [8], Ryten M.[8], Savage K.[8], Sawant K.[8], Scott R. H.[8], Siddiq A. [8] , Sieghart A. [8] , Smedley D[,42], Smith K. R. [8,42], Sosinsky A. [8,42], Spooner W. [8], Stevens H. E. [8], Stuckey A. [8], Sultana R. [8], Thomas E. R. A. [8,42], Thompson S. R.[8], Tregidgo C. [8], Tucci A. [8,42], Walsh E. [8], Watters, S. A. [8], Welland M. J. [8], Williams E. [8], Witkowska K. [8,42], Wood S. M. [8,42], Zarowiecki M.[8] .

8. Genomics England, London, UK

42. William Harvey Research Institute, Queen Mary University of London, London, UK
