# Supplementary material for: Genomic landscape of adult testicular germ cell tumours in the 100,000 Genomes Project
Source: Nat Commun. 2024 Oct 26;15:9247. doi: 10.1038/s41467-024-53193-6 (PMC11513037; doi:10.1038/s41467-024-53193-6)
Supplement: Supplementary file 3 — Description of Additional Supplementary Files [file 41467_2024_53193_MOESM3_ESM.pdf]

Description of Additional Supplementary Files

File Name: Supplementary Data 1
Description: Detailed information for Genomics England (GEL) Testicular Germ Cell Tumour (TGCT) samples and genomes reported in this study.

File Name: Supplementary Data 2
Description: List of nonsynonymous substitutions & indels called across the GEL TGCT cohort. Outputs from IntOGen-mutations pipeline.

File Name: Supplementary Data 3
Description: Outputs from GISTIC2 analysis.

File Name: Supplementary Data 4
Description: Outputs from analysis of structural variant clustering and hotspots.

File Name: Supplementary Data 5
Description: Outputs from AmpliconArchitect analysis.

File Name: Supplementary Data 6
Description: Outputs from mutational signatures analyses (SBS, DBS, ID, CN & SV signatures)

File Name: Supplementary Data 7
Description: Outputs from mutational timing analyses (WGD & focal amplification).

File Name: Supplementary Data 8
Description: Outputs from HLA analyses.
