# Supplementary material for: Genomic landscape of adult testicular germ cell tumours in the 100,000 Genomes Project
Source: Nat Commun. 2024 Oct 26;15:9247. doi: 10.1038/s41467-024-53193-6 (PMC11513037; doi:10.1038/s41467-024-53193-6)
Supplement: Supplementary file 12 — Reporting Summary [file 41467_2024_53193_MOESM12_ESM.pdf]

nature portfolio

Corresponding author(s): Máire Ní Leathlobhair, Andrew Protheroe, Matthew J Murray, David C Wedge, Clare Verrill

Last updated by author(s): August 15 2024

Reporting Summary

Nature Portfolio wishes to improve the reproducibility of the work that we publish. This form provides structure for consistency and transparency in reporting. For further information on Nature Portfolio policies, see our Editorial Policies and the Editorial Policy Checklist.

Statistics

For all statistical analyses, confirm that the following items are present in the figure legend, table legend, main text, or Methods section.

n/a	Confirmed	
☐	☒	The exact sample size (*n*) for each experimental group/condition, given as a discrete number and unit of measurement
☐	☒	A statement on whether measurements were taken from distinct samples or whether the same sample was measured repeatedly
☐	☒	The statistical test(s) used AND whether they are one- or two-sided<br>*Only common tests should be described solely by name; describe more complex techniques in the Methods section.*
☐	☒	A description of all covariates tested
☐	☒	A description of any assumptions or corrections, such as tests of normality and adjustment for multiple comparisons
☐	☒	A full description of the statistical parameters including central tendency (e.g. means) or other basic estimates (e.g. regression coefficient) AND variation (e.g. standard deviation) or associated estimates of uncertainty (e.g. confidence intervals)
☐	☒	For null hypothesis testing, the test statistic (e.g. *F*, *t*, *r*) with confidence intervals, effect sizes, degrees of freedom and *P* value noted<br>*Give P values as exact values whenever suitable.*
☐	☒	For Bayesian analysis, information on the choice of priors and Markov chain Monte Carlo settings
☐	☒	For hierarchical and complex designs, identification of the appropriate level for tests and full reporting of outcomes
☐	☒	Estimates of effect sizes (e.g. Cohen's *d*, Pearson's *r*), indicating how they were calculated

Our web collection on statistics for biologists contains articles on many of the points above.

Software and code

Policy information about availability of computer code

Data collection	Samples were collected and processed by Genomics England. All tumour-normal sample pairs were processed through 100,000 Genomes Project (100kGP) bioinformatic somatic-variant analysis pipelines. Further information regarding sequencing and alignment can be found in the Genomics England Cancer Analysis Technical Information Document Version 1.11 (https://files.genomicsengland.co.uk/forms/Cancer-Analysis-Technical-Information-Document-v1-11-main.pdf).
Data analysis	Details and code for using the Intogen pipeline are available at https://intogen.readthedocs.io/en/latest/index.html. The specific code used for this analysis, along with other methods described, is available in the Genomics England research environment under /re_gecip/shared_allGeCIPs/tgct_gecip/code/. The link to join the Genomics England research network and obtain data access can be found here https://www.genomicsengland.co.uk/research/academic/join-gecip.<br><br>Additional tools and packages used in this manuscript are listed below:<br>alleleCount-FixVAF (https://github.com/danchubb/alleleCount-FixVAF)<br>AmplificationTimeR (https://github.com/Wedge-lab/AmplificationTimeR)<br>Battenberg v2.2.7 (https://github.com/Wedge-Oxford/battenberg)<br>bcftools v1.9 (http://www.htslib.org/download/)<br>bedops v2.4.39 (https://github.com/bedops/bedops)<br>bedtools v2.3.0 (https://github.com/arq5x/bedtools2)<br>cBase (http://genetics.bwh.harvard.edu/wiki/sunyaevlab/cbase)<br>CleanCNA (https://github.com/afrangou/CleanCNA)<br>ClusterSV (https://github.com/cancerit/ClusterSV)<br>CNAqc (https://github.com/caravagnalab/CNAqc)

Delly v0.7.8 (https://github.com/dellytools/delly/releases/download/v0.7.9/delly_v0.7.9_linux_x86_64bit)
dNdSCV (https://github.com/im3sanger/dndscv)
DPClust v2.2.5 (https://github.com/Wedge-Oxford/dpclust)
FixVAF (https://github.com/danchubb/FixVAF)
GISTIC v2.0.2.3 (https://github.com/broadinstitute/gistic2)
HotMaps3D (https://github.com/KarchinLab/HotMAPS)
HRDetect (v0.0.0.9000 (https://github.com/Nik-Zainal-Group/signature.tools.lib)
igraph v1.2.4.2 (https://igraph.org/r/)
IntOGen (https://bitbucket.org/intogen/intogen-plus/src/master)
Isaac v03.16.02.19 (https://github.com/Illumina/Isaac3/releases/tag/iSAAC-03.16.02.19)
LOHHLA (https://bitbucket.org/mcgranahanlab/lohhla/src/master/)
Lumpy v0.2.13 (https://github.com/arq5x/lumpy-sv/releases/download/0.2.13/lumpy-sv-v0.2.13.tar.gz)
Manta v0.28.0 (https://github.com/Illumina/manta/releases/download/v0.28.0/manta-0.28.0.release_src.tar.bz2)
MuTect v1.16 (https://software.broadinstitute.org/cancer/cga/mutect_download)
MutPanning (https://github.com/vanallenlab/MutPanningV2)
MutationTimeR (https://github.com/gerstung-lab/MutationTimeR)
OncodriveCLUSTL (https://bitbucket.org/bbglab/oncodriveclustl/src/master/)
OncodriveFML (https://bitbucket.org/bbglab/oncodrivefml/src/master)
PCAWG-11 for chronological WGD (https://gerstung-lab.github.io/PCAWG-11/)
PCAWG SV merge (https://hub.docker.com/r/weischenfeldt/pcawg_sv_merge)
POLYSOLVER (https://software.broadinstitute.org/cancer/cga/polysolver_download)
PlackettLuce (https://github.com/hturner/PlackettLuce)
R v3.6.2 and 4.0.2 (https://cran.ma.imperial.ac.uk/)
SigProfilerExtractor v1.1.3 (https://github.com/AlexandrovLab/SigProfilerExtractor/releases/tag/v1.1.3)
SigProfilerMatrixGenerator (https://github.com/AlexandrovLab/SigProfilerMatrixGenerator)
smRegions (https://bitbucket.org/bbglab/smregions/src/master/)
Strelka v2.9.9 (https://github.com/Illumina/strelka/releases/tag/v2.9.9)
trackViewer (https://github.com/jianhong/trackViewer)
UTRannotator (https://github.com/ImperialCardioGenetics/UTRannotator)
VEP (https://www.ensembl.org/info/docs/tools/vep/script/vep_download.html)
xTea (https://github.com/parklab/xTea)

For manuscripts utilizing custom algorithms or software that are central to the research but not yet described in published literature, software must be made available to editors and reviewers. We strongly encourage code deposition in a community repository (e.g. GitHub). See the Nature Portfolio guidelines for submitting code & software for further information.

Data

Policy information about availability of data

All manuscripts must include a data availability statement. This statement should provide the following information, where applicable:

- Accession codes, unique identifiers, or web links for publicly available datasets
- A description of any restrictions on data availability
- For clinical datasets or third party data, please ensure that the statement adheres to our policy

Data summary statistics are provided in the Supplementary Data where such data does not enable identification of participants. Primary data from the 100,000 Genomes Project, which are held in a secure Research Environment, are available to registered users. Users must be affiliated with a registered institution and must have applied to join the Pan-Cancer and Molecular Oncology community (https://www.genomicsengland.co.uk/research/pan-cancer-and-molecular-oncology-community). The process involves an online application, verification by the applicant's institution, completion of a short information governance training course, and verification of approval by Genomics England. Please see https://www.genomicsengland.co.uk/research/academic/join-gecip for more information. All analysis of Genomics England data must take place within the Genomics England Research Environment (https://re-docs.genomicsengland.co.uk/). Users will be given access to the information and data held in the Research Environment whilst they remain members. An individual's membership is renewed automatically each year unless the Office of the Chief Scientist notifies the individual that their membership has not been renewed.
The 100,000 Genomes Project publication policies can be obtained from https://www.genomicsengland.co.uk/research/publications/publication-policy. Results used in this study are provided in Genomics England under /re_gecip/shared_allGeCIPs/tgct_gecip/results/.

See https://www.genomicsengland.co.uk/research/academic for more information or contact the Chief Scientific Officer at Genomics England.

Public datasets used in this study include the following:
- MSK-MET TGCT dataset (Nguyen et al., Cell 2022) downloaded from the cBio Portal for Cancer Genomics (https://www.cbioportal.org/study/summary?id=msk_met_2021)
- TCGA TGCT dataset (Shen et al., Cell Reports 2018) downloaded from cBioPortal for Cancer Genomics (https://www.cbioportal.org/study/summary?id=tgct_tcga_pan_can_atlas_2018)
- OncoKB v3.3 (http://oncokb.org/)
- IntOGen Gene Annotations (https://www.intogen.org/download?file=IntOGen-Cohorts-20200201.zip)
- Homo Sapiens GRCh38Decoy reference assembly (http://emea.support.illumina.com/sequencing/sequencing_software/igenome.html)
- Protein Data Bank (https://www.rcsb.org/#Category-download)
- GnomAD v2.1 (https://gnomad.broadinstitute.org/downloads#v2-constraint)
- Ensembl 101 (https://www.ensembl.org/index.html)
- ENCODE v101 (https://www.encodeproject.org)
- Replication Domain (https://www2.replicationdomain.com)
- CADD v1.6 (https://cadd.gs.washington.edu)
- CancerMine (http://bionlp.bcgsc.ca/cancermine/)
- COSMIC Cancer Gene Census v92 (https://cancer.sanger.ac.uk/census)
- COSMIC Reference Mutational Signatures v3.2 (https://cancer.sanger.ac.uk/signatures/)
- Segmental Duplication Database (https://humanparalogy.gs.washington.edu)

Research involving human participants, their data, or biological material

Policy information about studies with human participants or human data. See also policy information about sex, gender (identity/presentation), and sexual orientation and race, ethnicity and racism.

Reporting on sex and gender	Participants recruited to the study included anyone with a likely diagnosis of a testicular tumour, regardless of sex or gender, in accordance with SAGER guidelines. The sex of participants was reported by the submitting clinical team and was not determined by our research team.
Reporting on race, ethnicity, or other socially relevant groupings	Reported race, ethnicity, or other socially relevant groupings were not used in this study.
Population characteristics	Information relating to the cohort in this analysis are provided in the Supplementary datasets. The collection and processing of treatment information is described in detail in the Methods and Supplementary Methods.
Recruitment	Participants were recruited to the 100,000 Genomes Project in accordance to the Declaration of Helsinki ethical principles for medical research. Recruitment was coordinated by clinicians in seven GMCs across England. Locations of these GMCs are detailed in the manuscript. Clinical and demographic data were obtained from NHS Digital (NHSD), Public Health England's National Cancer Registration and Analysis Service (PHE-NCRAS) and GMCs via the Genomics England Research Environment.
Ethics oversight	All participants provided written informed consent. Genomics England has approval from the Health Research Authority Committee East of England – Cambridge South (REC Ref 14/EE/1112). Additional ethical oversight is provided by the Genomics England's Ethics Advisory Committee and Participant Panel.

Note that full information on the approval of the study protocol must also be provided in the manuscript.

Field-specific reporting

Please select the one below that is the best fit for your research. If you are not sure, read the appropriate sections before making your selection.

☒ Life sciences ☐ Behavioural & social sciences ☐ Ecological, evolutionary & environmental sciences

For a reference copy of the document with all sections, see nature.com/documents/nr-reporting-summary-flat.pdf

Life sciences study design

All studies must disclose on these points even when the disclosure is negative.

Sample size	No sample size calculation was performed. 60 samples were included in the final cohort. Sample size was chosen based on the availability of whole genome sequencing of suitable (see Data exclusions) tumour/normal pairs in the Genomics England research environment.
Data exclusions	Two samples were excluded from the available data. This included one case which was a Diffuse B-Cell Lymphoma that had been misclassified as a TGCT Disease Type and one FFPE sample type. In cases where inclusion of multiple samples from a single individual would confound analysis, these were excluded and only one of the multi-samples with the highest tumour purity was used.
Replication	Not applicable. This study has an observational rather than an experimental study design and, apart from one participant that underwent multi-region sampling, all data derived from different individuals. Technical replicates were not needed as sequencing noise and artefacts were accounted for using bioinformatic approaches.
Randomization	This study is a non-interventional and descriptive analysis of genomic data made available via NHS England. Randomization was therefore not applicable.
Blinding	This study is a non-interventional and descriptive analysis of genomic data made available via NHS England. Blinding was therefore not required. Investigators did not have control over sample selection and as such blinding is not relevant to this study

Reporting for specific materials, systems and methods

We require information from authors about some types of materials, experimental systems and methods used in many studies. Here, indicate whether each material, system or method listed is relevant to your study. If you are not sure if a list item applies to your research, read the appropriate section before selecting a response.

Materials & experimental systems

n/a	Involved in the study
☒	☐ Antibodies
☒	☐ Eukaryotic cell lines
☒	☐ Palaeontology and archaeology
☒	☐ Animals and other organisms
☒	☐ Clinical data
☒	☐ Dual use research of concern
☒	☐ Plants

Methods

n/a	Involved in the study
☒	☐ ChIP-seq
☒	☐ Flow cytometry
☒	☐ MRI-based neuroimaging

Plants

Seed stocks	*Report on the source of all seed stocks or other plant material used. If applicable, state the seed stock centre and catalogue number. If plant specimens were collected from the field, describe the collection location, date and sampling procedures.*
Novel plant genotypes	*Describe the methods by which all novel plant genotypes were produced. This includes those generated by transgenic approaches, gene editing, chemical/radiation-based mutagenesis and hybridization. For transgenic lines, describe the transformation method, the number of independent lines analyzed and the generation upon which experiments were performed. For gene-edited lines, describe the editor used, the endogenous sequence targeted for editing, the targeting guide RNA sequence (if applicable) and how the editor was applied.*
Authentication	*Describe any authentication procedures for each seed stock used or novel genotype generated. Describe any experiments used to assess the effect of a mutation and, where applicable, how potential secondary effects (e.g. second site T-DNA insertions, mosiacism, off-target gene editing) were examined.*
